# Emerging nanotechnology-based therapeutics to combat multidrug-resistant cancer

**DOI:** 10.1186/s12951-022-01626-z

**Published:** 2022-09-24

**Authors:** Priya Yadav, Suresh V. Ambudkar, N. Rajendra Prasad

**Affiliations:** 1 Department of Biochemistry and Biotechnology, Annamalai University, Annamalainagar, Tamil Nadu 608 002 India; 2 Laboratory of Cell Biology, Center for Cancer Research, National Cancer Institute, National Institutes of Health, 37 Convent Drive, Bethesda, MD 20892-4256 USA

**Keywords:** Multidrug resistance, Nanotechnology, P-glycoprotein, ABC transporter, Combinational therapy, Tumor microenvironment, Cancer stem cells, Drug delivery

## Abstract

Cancer often develops multidrug resistance (MDR) when cancer cells become resistant to numerous structurally and functionally different chemotherapeutic agents. MDR is considered one of the principal reasons for the failure of many forms of clinical chemotherapy. Several factors are involved in the development of MDR including increased expression of efflux transporters, the tumor microenvironment, changes in molecular targets and the activity of cancer stem cells. Recently, researchers have designed and developed a number of small molecule inhibitors and derivatives of natural compounds to overcome various mechanisms of clinical MDR. Unfortunately, most of the chemosensitizing approaches have failed in clinical trials due to non-specific interactions and adverse side effects at pharmacologically effective concentrations. Nanomedicine approaches provide an efficient drug delivery platform to overcome the limitations of conventional chemotherapy and improve therapeutic effectiveness. Multifunctional nanomaterials have been found to facilitate drug delivery by improving bioavailability and pharmacokinetics, enhancing the therapeutic efficacy of chemotherapeutic drugs to overcome MDR. In this review article, we discuss the major factors contributing to MDR and the limitations of existing chemotherapy- and nanocarrier-based drug delivery systems to overcome clinical MDR mechanisms. We critically review recent nanotechnology-based approaches to combat tumor heterogeneity, drug efflux mechanisms, DNA repair and apoptotic machineries to overcome clinical MDR. Recent successful therapies of this nature include liposomal nanoformulations, cRGDY-PEG-Cy5.5-Carbon dots and Cds/ZnS core–shell quantum dots that have been employed for the effective treatment of various cancer sub-types including small cell lung, head and neck and breast cancers.

**Graphical Abstract:**

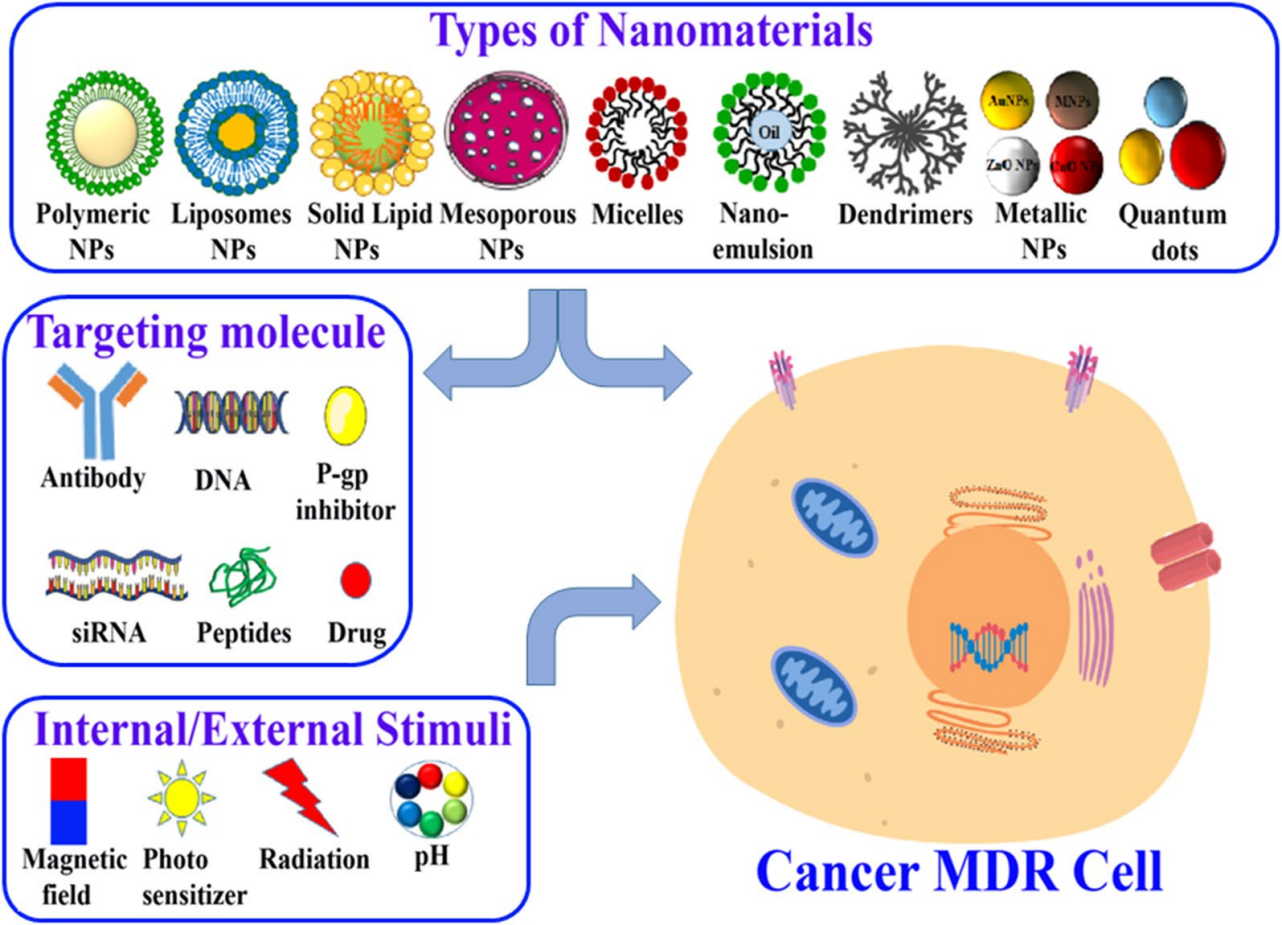

## Background

Cancer is a deadly disease characterized by the uncontrolled proliferation of cells. Mutations followed by genetic instabilities result in the initiation, progression and development of tumors [1]. Cancer is one of the leading causes of death globally, accounting for 10 million deaths in 2020 [2]. The main treatment modalities to eradicate different sub-types of cancers are surgery, radiotherapy, chemotherapy, hormone therapy and immunotherapy or a combination of these therapies. Various reports illustrate that chemotherapy often fails in the clinic and accounts for more than 25% of mortality in cancer patients [3–5].

Multidrug resistance (MDR) mechanisms limit the efficacy of chemotherapy in cancer cells [6–8] and have been considered some of the most challenging obstacles to effective chemotherapy [8, 9]. The reoccurrence of tumors and associated relapse or deaths of cancer patients are mainly attributable to either the intrinsic or acquired phenomenon of MDR. Some cancer cells are inherently unresponsive to certain anticancer drugs [9]. Others acquire resistance to chemotherapy during the course of chemotherapy. This acquired MDR phenomenon is mainly due to repetitive exposure to chemotherapeutic drugs [10]. The ATP-binding cassette (ABC) drug efflux transporters such as P-glycoprotein (P-gp; ABCB1; MDR1), ABCG2 (also called breast cancer resistance protein, BCRP) and MRP-1 (ABCC1) are often overexpressed after the initial treatment regimen [7, 11].

## Mechanisms associated with MDR in cancer

**Fig. 1:**
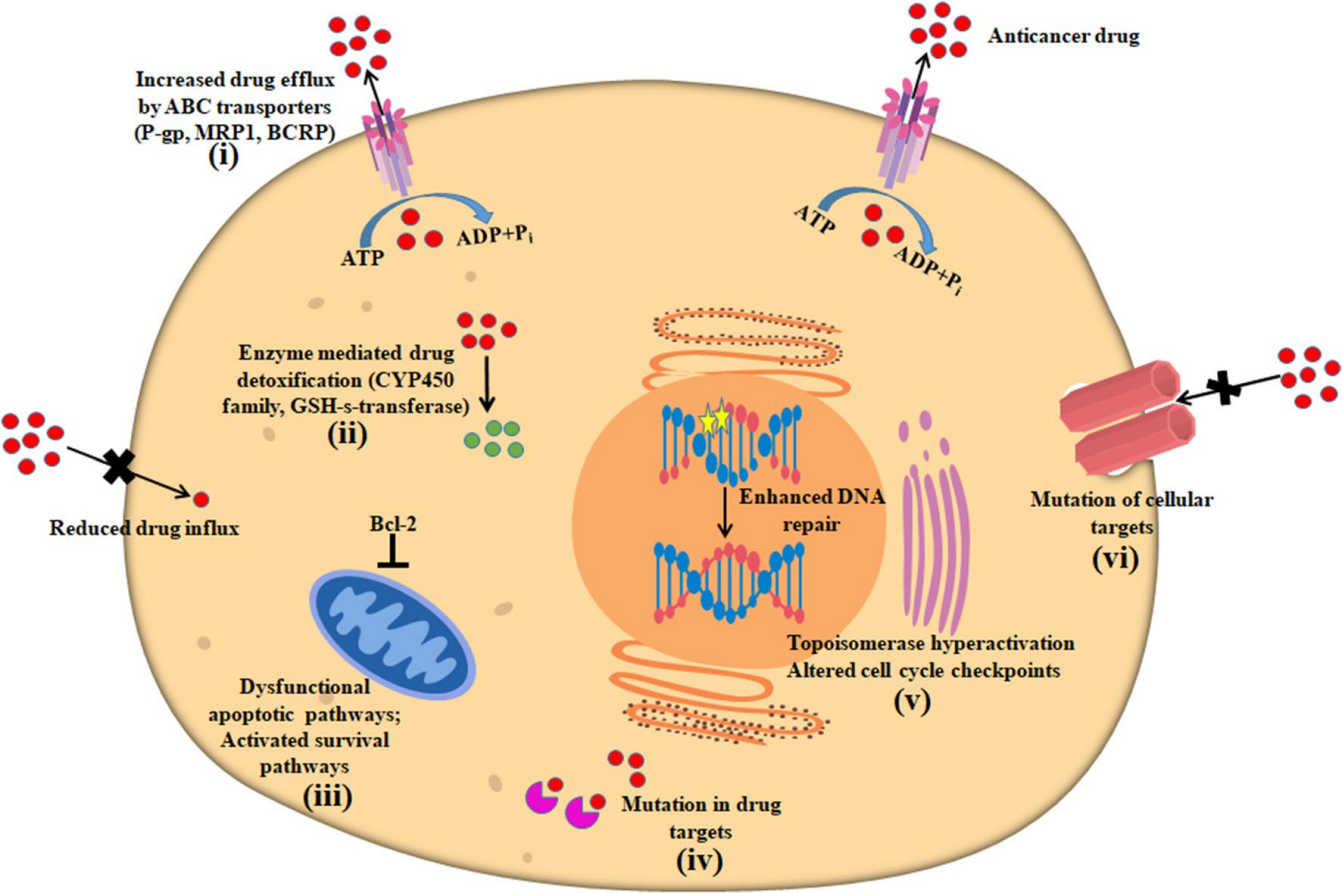
Mechanisms contributing to the development of MDR in cancer cells. Various mechanisms such as (i) increased drug efflux by ABC drug transporters, (ii) inactivation of drugs via cellular metabolism and detoxification, (iii) dysfunctional apoptotic pathways, (iv) mutations in drug targets, (v) enhanced DNA repair mechanisms and (vi) mutations in cellular targets play roles in the development of cancer MDR

The phenomenon of MDR is a complex and multifactorial process, illustrated in Fig. 1. MDR arises due to various mechanisms including overexpression of ABC transporters that efflux chemotherapeutics [12], mutations in drug targets [8], the developing adaptation of cancer cells to the microenvironment, and increased efflux of hydrophobic chemotherapeutic drugs. The alteration of drug targets either due to epigenetic changes or secondary mutations in the target protein can result in multidrug-resistant cancer [8]. Principally, cancer cells develop MDR by overexpressing drug efflux transporters [13]. ABC drug transporters energetically fueled by ATP hydrolysis are responsible for the low bioavailability of chemotherapeutic drugs [14, 15]. Cancer cells dynamically adapt to the changing microenvironment. For example, increased oxidative stress contributes to tumor development, and DNA mutations can lead to MDR [16]. Therefore, dynamic activation of the DNA repair system in tumor cells also contributes to MDR [17, 18]. Enhanced DNA repair pathways and chromatin dynamics are known to be associated with the development of MDR in tumor cells [9, 17, 19]. One recent clinical study illustrated the impact of DNA repair on genomic stability and resistance to the anticancer drug treatment of pediatric high-grade gliomas [20]. Cancer cells also become accustomed to hypoxic tissue conditions by overexpression of hypoxia-inducible factor-1α (HIF-1 α). Hypoxia triggers cancer MDR by reducing the efficacy of chemotherapeutic drugs. It may also stimulate the expression of ABC transporter pumps that eventually efflux intracellular chemotherapeutic drugs [21].

Dysfunctional apoptotic pathways, increased repair of DNA damage, alterations in the cell cycle, and overexpression of cyclin-dependent kinases (CDKs) contribute to the development of resistance to chemotherapeutic drugs in cancer cells [22]. Moreover, defective apoptotic machinery has been associated with treatment failure in cancer clinics. For example, mutations in the p53 tumor suppressor gene or disrupted functions of p53 protein have been found to be responsible for treatment failure and poor prognosis in B- and T-cell Non-Hodgkin’s lymphoma [23, 24]. Rapid metabolism of anticancer drugs and detoxification of drugs by cytochrome P450 are associated with rapid turnover and elimination of anticancer drugs [25]. Therefore, inactivation and detoxification of chemotherapeutic drugs by human cytochrome P450s (CPY) phase I and/or II enzymes can contribute to the development of cancer MDR [7]. A recent report demonstrated that the inter-individual variation in cytochrome P450 expression determines the chemotherapeutic drug efficacy [26]. Furthermore, tumor heterogeneity plays a major role in the development of MDR [27, 28] as cancer stem cells (CSCs) are capable of self-renewal and differentiation [29]. Table 1 shows various ways nanoparticles have been used to combat cancer MDR.

**Table 1:** Various applications of nanoparticles to combat cancer MDR

Target	Chemotherapeutic agent	Mechanism of action	Type of nanoparticles	Refs
Efflux transporters	P-gp targeted siRNA and/or P-gp inhibitors	Bypass and/or inhibit efflux transporter	Polymeric NPs	[217–220]
			Lipid NPs	[77, 221, 222]
			Silicon NPs	[141, 223, 224]
			Gold NPs	[133, 225–227]
			Graphene oxide NPs	[228–230]
Hypoxia	HIF-1α siRNA	Silence HIF-1α gene	Lipid NPs	[231–233]
			Micellar NPs	[149, 174, 234]
			Polymeric NPs	[175, 235, 236]
	HIF-1α inhibitors (PX-478)	Inhibit the function of HIF-1α	SPION NPs	[237, 238]
			Silver NPs	[173, 239]
			Cu2-xSe NPs	[240]
Apoptosis	Bcl-2-targeted siRNA	Inhibit anti-apoptotic pathway	SPION NPs	[241]
			Mesoporous silica NPs	[204]
			Polymeric NPs	[242–244]
	NF-κB inhibitor	Activate pro-apoptotic pathway	Polymeric NPs	[156, 245, 246]
Cell cycle	Flavopiridol, siRNA and UCN-01	Inhibit CDK	Polymeric NPs	[247–253]
			Metallic NPs	[254]
Detoxification system	Buthionine sulfoximine (BSO)	Inhibit GSH biosynthesis	Polymeric NPs	[255, 256]
			Metal NPs	[257, 258]
	Ethacrynic acid	Inhibit GST	Metal NPs	[259]
			Polymeric NPs	[193, 260]

### Multidrug resistance and ABC drug efflux transporters

Some of the members of the superfamily of ABC proteins are typically expressed on the plasma membrane. They efflux cytotoxic agents from cells, thereby contributing to clinical MDR [30–33]. ABC transporters play a major role in the absorption, distribution, metabolism, excretion and toxicity (ADMET) of drugs [32]. Mammalian P-gp is the most widely studied transporter and it plays a significant role in MDR [34].

Since the early 1990s many drugs have been evaluated for their possible inhibition of ABC efflux transporters. First-generation P-gp inhibitors such as verapamil, cyclosporine A, quinine, and erythromycin were found to be effective in-vitro but showed inadequate pharmacological limitations, adverse side effects and low affinity towards this transporter during in-vivo experiments [35, 36]. To prevail over the adverse side effects of first-generation inhibitors, researchers modified their structures and these inhibitors, known as second-generation P-gp inhibitors, were developed including dexverapamil, S9788, and PSC-833 also called valspodar (cyclosporine A analog), etc. The second-generation P-gp inhibitors often caused interference with anticancer drugs and affected their pharmacokinetics, resulting in adverse side effects [37, 38]. The third generation of inhibitors such as elacridar, zosuquidar and tariquidar were subsequently tested in clinical studies but also failed to achieve clinical approval due to severe cytotoxic side effects [39, 40].

Fourth-generation inhibitors include natural compounds and several flavonoids with inhibitory effects on ABC efflux pumps. Natural compounds such as curcumin, piperine, tea polyphenol epigallocatechin-3-gallate (EGCG), silibinin, parthenolide, quercetin, capsaicin, carnosic acid, 6-gingerol, procyanidin, limonin, and β-carotene act as inhibitors of P-gp, and can be utilized as chemosensitizing agents to reverse MDR. Natural phytochemicals can sometimes downregulate P-gp expression by modulating different cell signaling pathways. These phytochemicals augment chemotherapy-mediated apoptotic signals in P-gp-overexpressing cells [41]. They have been found to alter the MAPK, PI3K, and GSK signaling pathways that promote the activation of downstream signaling molecules such as AP-1, NF-κB and β-catenin. These signaling molecules interact with transcription factors and initiate the downregulation of P-gp in cancer cells, eventually assisting in the reversal of P-gp-mediated MDR. In one study, for example, Ganesan et al*.* demonstrated the role of ferulic acid on P-gp modulation to overcome MDR in colchicine-selected KB-Ch^R^-8–5 resistant cells and in the MDR xenograft mouse model via the PI3K/Akt/NF-κB signaling pathway [42]. These natural compounds were established as potential candidates with no toxicity but did not succeed due to minimal solubility and bioavailability, hampering their efficacy. Therefore, they could not be established as potent P-gp inhibitors or successful contenders to reverse chemoresistance [43, 44].

### Tyrosine kinase inhibitors as modulators of drug efflux transporters

More than 50 tyrosine kinase inhibitors (TKIs) have been found to be efficient in clinical research and are approved by the US Food and Drug Administration (FDA) for anticancer therapy [45, 46]. Numerous investigations indicated that TKIs in addition to their kinase target also interact with the ABC efflux pumps [47, 48]. These inhibitors were found to competitively bind at the drug-substrate-binding site of the ABC efflux pumps, thereby inhibiting their function and sensitizing the drug-resistant cancer cells. This chemosensitization enhances the intracellular accumulation of drugs in cancer cells. The first generation TKI imatinib reverses the ABCG2-mediated chemoresistance of topotecan [49] and doxorubicin [50] in experimental models. Another inhibitor, dacomitinib, was shown to inhibit ABCG2 efflux pumps and enhance drug accumulation and retention, thereby reversing ABCG2-mediated MDR in cancer cells [51]. Combination treatment of dacomitinib and topotecan appreciably inhibits tumor growth as compared to topotecan and/or dacomitinib treatment alone, without any additional toxicity. Narayanan et al*.* performed an extensive in-vitro study that tested the role of the spleen TKI entospletinib (GS-9973) in the reversal of ABCG2-mediated MDR. Entosletinib was found to reverse resistance to mitoxantrone and doxorubicin in cells overexpressing ABCG2 transporters. The ATPase activity of ABCG2 was enhanced due to the binding of entospletinib at the drug-substrate binding site [52]. Yang et al*.* reported that sitravatinib interferes with the tumor microenvironment and immune-checkpoint blockade (PD-1) in many cancer models [53]. It also has the potency to reverse MDR mediated by the ABCG2 efflux pump in cancer cells. Combination therapies along with FDA-approved TKIs and established chemotherapeutics are under clinical trials [54, 55]. Major drawbacks of using TKIs as adjuvants with chemotherapy are their poor solubility, adverse toxicity and severe side effects in patients [56, 57].

### Small interfering RNA (siRNA) for inhibition of drug efflux transporters

Combining gene therapy with chemotherapeutic agents can sometimes improve therapeutic efficacy. Various types of nucleic acid-based molecules such as small interfering RNAs (siRNAs), plasmid DNA, short hairpin loops and circulating miRNAs enable the regulation of specific genes to regulate and reverse MDR in cancer cells [58, 59]. Donmez and co-workers sensitized resistant breast cancer cells by transfecting with *MDR1* siRNA plus doxorubicin to overcome P-gp-mediated cancer MDR. The siRNA targeting the *MDR1* gene successfully silenced the *MDR1* mRNA by approximately 90% and enhanced the accumulation of doxorubicin in drug-resistant cells [60]. Major obstacles to applying nucleic acid-based drugs are their stability, enzymatic degradation, poor membrane permeability and short half-life.

## Nanotechnology-based strategies to overcome MDR

To overcome the inadequacies of existing treatment and therapy, nanomedicine offers innovative, robust and flexible drug design and delivery alternatives based on genetic profiling of individual patients to engender personalized treatment of cancer MDR [61–63]. The fascinating physicochemical properties of nanomaterials contribute to the improvement of the therapeutic index of potential chemotherapeutic drugs by enhancing their efficacy and reduced adverse toxic effects. Multimodal nanoformulations composed of materials such as gold, iron or quantum dots, functionalized with ABC efflux pump inhibitors and targeting molecules/peptides, have been shown to improve the pharmacokinetics and biodistribution of chemotherapeutic drugs in multidrug-resistant cancer cells [64]. P-gp inhibitors released in cancer cells from nanocarriers bind at the drug-binding pocket in the transmembrane domains (TMDs) of the transporters and inhibit their drug efflux function [65]. This approach was reported to enhance the therapeutic efficacy of several anticancer drugs [66–68]. Similarly, the co-delivery of suitable adjuvants using nanocarriers can improve the anticancer drugs' therapeutic efficacy by targeting the drug detoxification process, DNA repair mechanism and apoptotic cell death [52, 69, 70].

The delivery of nanomaterials to tumor cells is typically achieved by both active and passive mechanisms. In the active mode of nanoparticle uptake, the surface of nanoparticles is decorated with specific targeting ligands such as antibodies or peptides, cell-specific ligands which facilitate uptake of the nanoparticles via receptor-mediated endocytosis. During passive uptake, the nanomaterials tend to accumulate in the tumor interstitial spaces due to long-circulating systemic properties and are selectively taken up by cells due to leaky vasculature and impaired lymphatic systems [71]. Passive uptake is mainly achieved by the enhanced permeability and retention (EPR) effect in cancer cells [72]. The co-delivery of inhibitors of ABC efflux transporters and potent anticancer chemotherapeutic drugs via nanocarriers has been widely explored, accepted and is under clinical investigations to overcome MDR in tumors [73].

Various nanomaterials found successful for drug delivery and targeting tumors are liposomes, polymeric nanoparticles, micelles, dendrimers, metal nanoparticles, mesoporous silica nanoparticles, graphene nanoparticles, quantum dots and siRNA-conjugated nanomaterials, which all help to reverse the MDR in cancer cells. Dual drug delivery via nanoparticle systems was also developed in which combinations of drugs are co-delivered to cells, and the presence of one drug enhances the bioavailability of another drug [74, 75]. Certain non-ionic surfactants have been investigated for the inhibition of ABC efflux transporters and reversal of MDR, including polyethylene glycol, Tween 80, and Pluronics. These surfactants are known to evade recognition by P-gp, facilitating the intracellular uptake of drugs. Besides surfactants, other nanoformulations such as liposomes, polymeric nanoparticles, metallic nanoparticles, nanoemulsions, and inorganic nanoparticles have been designed with the ability to bypass drug efflux transporters and deliver chemotherapeutic drugs to MDR cancer cells [75–80].

**Fig. 2:**
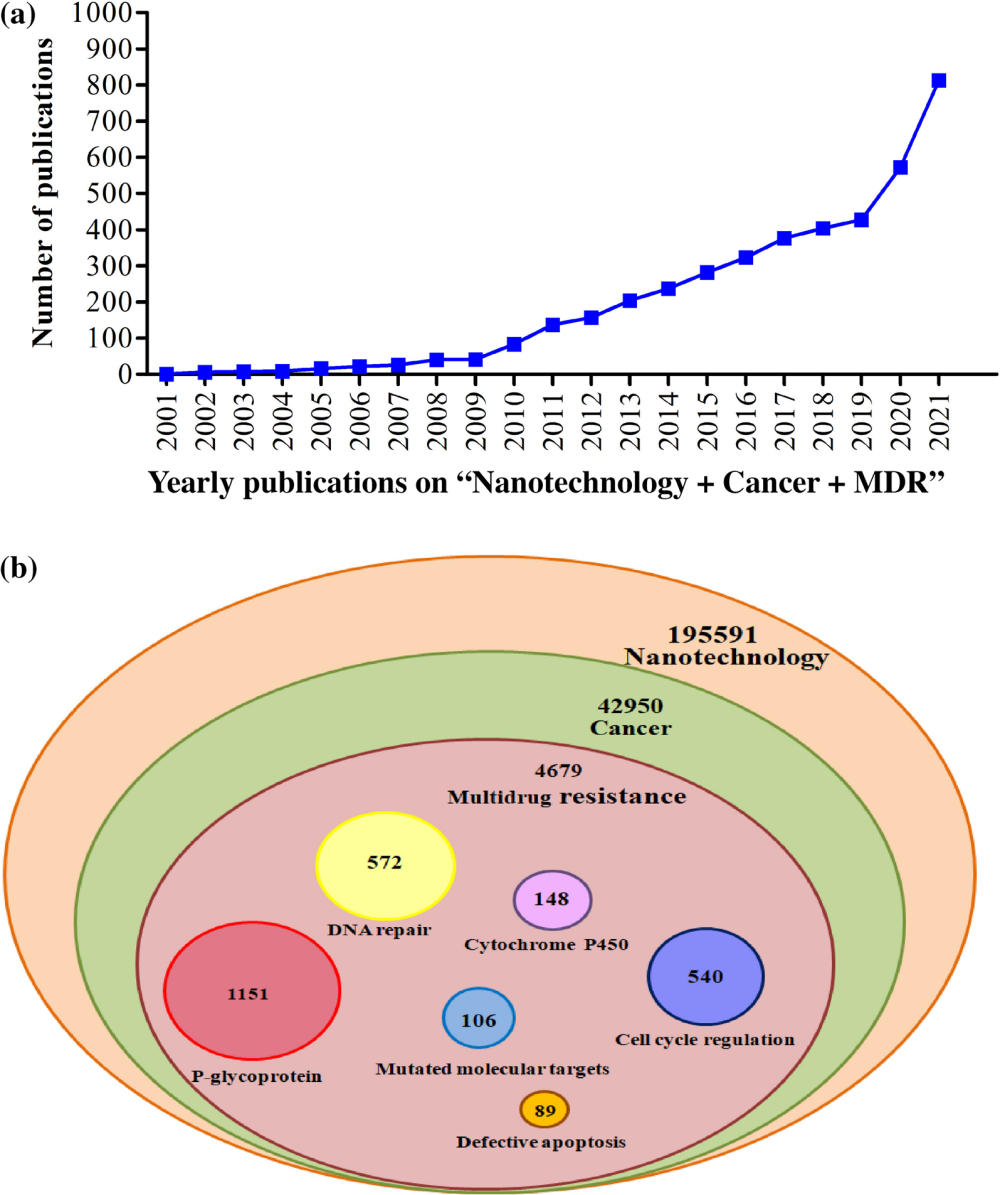
Scopus published research articles on nanomediated approaches to overcome MDR in cancer. **a** Graph showing the total number of articles published with the keywords “Nanotechnology”, “Cancer” and “Multidrug resistance”. The results show increasing interest in the nanomedicine approach to overcome MDR in cancer. **b** Venn diagram categorizing articles containing the keywords “Nanotechnology”, “Cancer” and “Multidrug resistance” and their combination with different keywords such as P-glycoprotein, DNA repair, Cytochrome P450, Defective apoptosis, Cell cycle regulation and Mutated molecular targets

Furthermore, the combination of chemotherapeutic drugs with gene therapy, specifically siRNA co-delivery via nanoparticles, was found to be more successful in the reversal of cancer MDR by targeting cellular signaling pathways [81, 82]. Nanocarriers provide stability to siRNA, thereby preventing its rapid degradation and clearance in the cellular system [83]. Anselmo et al*.* provided an update on several nanoparticles which showed improved therapeutic abilities in clinical studies and listed the approval status of promising nanosystems to improve human health from the early 1990s to 2019 [84]. In recent years, nanosystems have gained more attention for the delivery of chemotherapeutic drugs with suitable adjuvants to circumvent MDR in different cancer subtypes. Figure 2a shows the number of research articles published during the years 2001–2021 on the reversal of cancer MDR in various experimental models via chemotherapeutic drugs and/or adjuvant-conjugated nanomaterials. In the last 10 years, the number of such articles has quadrupled. The Venn diagram in Fig. 2b categorizes a total of 195,591 published research articles on nanotechnology. More than 42,950 of them involved cancer research, with 4679 (10%) of the nanotechnology and cancer articles specifically dealing with multidrug resistance.

ABC transporters are overexpressed by brain endothelial cells that form the blood–brain barrier (BBB) and are involved in the efflux of toxic foreign compounds as well as blood-derived compounds. These transporters prevent chemotherapeutic drugs from reaching their target site of action within the brain [85, 86]. Several polymeric, liposome-based and metallic nanoformulations were found to be suitable carriers to cross the BBB for controlled and sustained drug delivery. The surfaces of these nanoformulations were modified to enable them to cross the BBB for accurate diagnosis and to deliver appropriate anticancer drugs to treat brain tumors [87, 88]. Gregory et al*.* reported the efficacy of iRGD functionalized albumin-based synthetic protein nanoparticles (SPNPs) to deliver siRNA specific for STAT3 into intracranial GBM tumors. STAT3 siRNA-loaded SPNPs showed efficient penetration of the BBB, significant downregulation of the STAT3 expression and tumor regression in both GL26 glioma cell and GL26 syngeneic mouse models [89]. The use of transferring receptor (TR)-targeted liposomal nanoformulation was found to significantly enhance the delivery of cisplatin across the BBB for the treatment of brain tumors in C6 cells and Wistar rats [90].

**Fig. 3:**
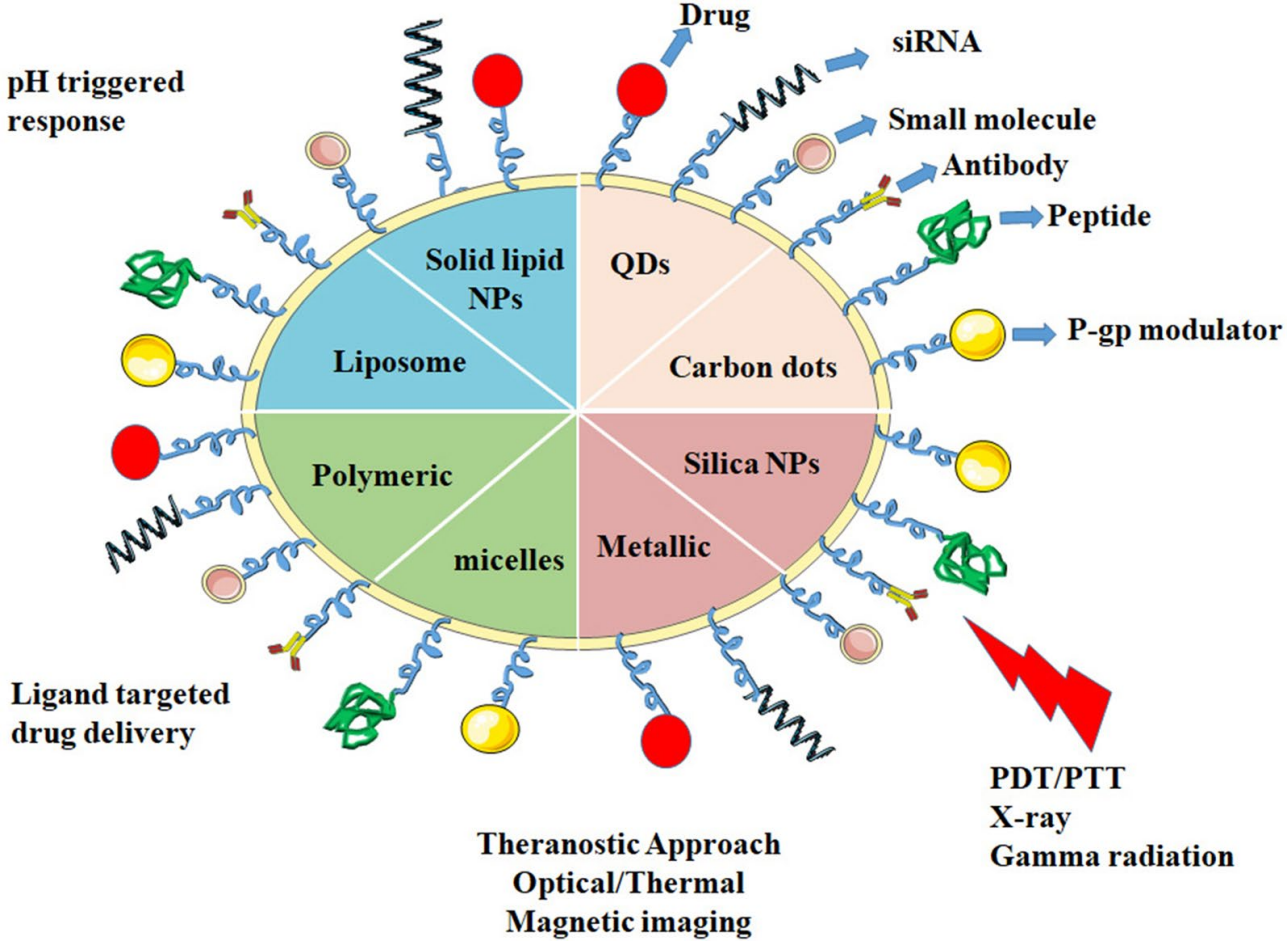
Strategies using various nanomaterials functionalized and surface modified with appropriate ligands that reach the target site and deliver the drug to overcome MDR**.** Active targeting and transporting the drugs to the tumor site allow nanosystems to inhibit the efflux of proteins, modulate the expression of anti-apoptotic genes and also enhance intracellular drug retention based on responses to different stimuli (light, X-rays, and gamma-rays). Metallic nanosystems facilitate the optical, thermal, and magnetic imaging of solid tumors

Over the past decade, photodynamic therapy (PDT) has attracted substantial attention as an efficacious alternative treatment approach to overcome MDR. Delivery of photosensitizers and drugs simultaneously is difficult. It was found that PDT could also be improved by employing nanomaterials to mitigate MDR [91]. PDT mainly eradicates cancer cells through the transfer of energy from light-activated photosensitizers to oxygen and generates intracellular oxidative stress via reactive oxygen species (ROS) [92]. The resultant intracellular ROS decreases the expression of membrane efflux proteins and anti-apoptotic Bcl-2 family proteins [93]. Due to disruption of mitochondrial membranes, the level of intracellular ATP declines and the activity of ATP-dependent ABC proteins is subsequently decreased. Guo and co-workers revealed the use of a nanosized hydrogel-like polyprodrug of platinum (IV) complex that has long-term circulation, tumor accumulation and also generates a high level of intracellular ROS. The elevated level of ROS downregulates the expression of MDR-associated protein 1 (MRP1), thus reversing MDR in A549R cells and in A549 tumor-bearing BALB/c mice model [94]. Li et al*.* demonstrated the role of mitoxantrone loaded poly (ε-caprolactone)-pluronic F68-poly (ε-caprolactone)/PLGA-PEG-PLGA) mixed nanomicelles to reverse MDR in MCF-7/ADR cells under exposure of near-infrared (NIR) light. These nanomicelles upon irradiation with NIR light generate higher levels of ROS, thus decreasing P-gp activity, leading to improved, higher concentrations of intracellular drugs and further cell apoptosis. This approach reverses MDR via nano-mediated PDT [95]. Figure 3 summarizes the strategies of different multimodal nanosystems functionalized with various targeting molecules to deliver drugs. These nanosystems are able to reverse MDR under the influence of various stimuli depending on the tumor microenvironment.

## Nanocarrier-based drug delivery systems to overcome MDR

### Polymeric nanomaterials

**Fig. 4:**
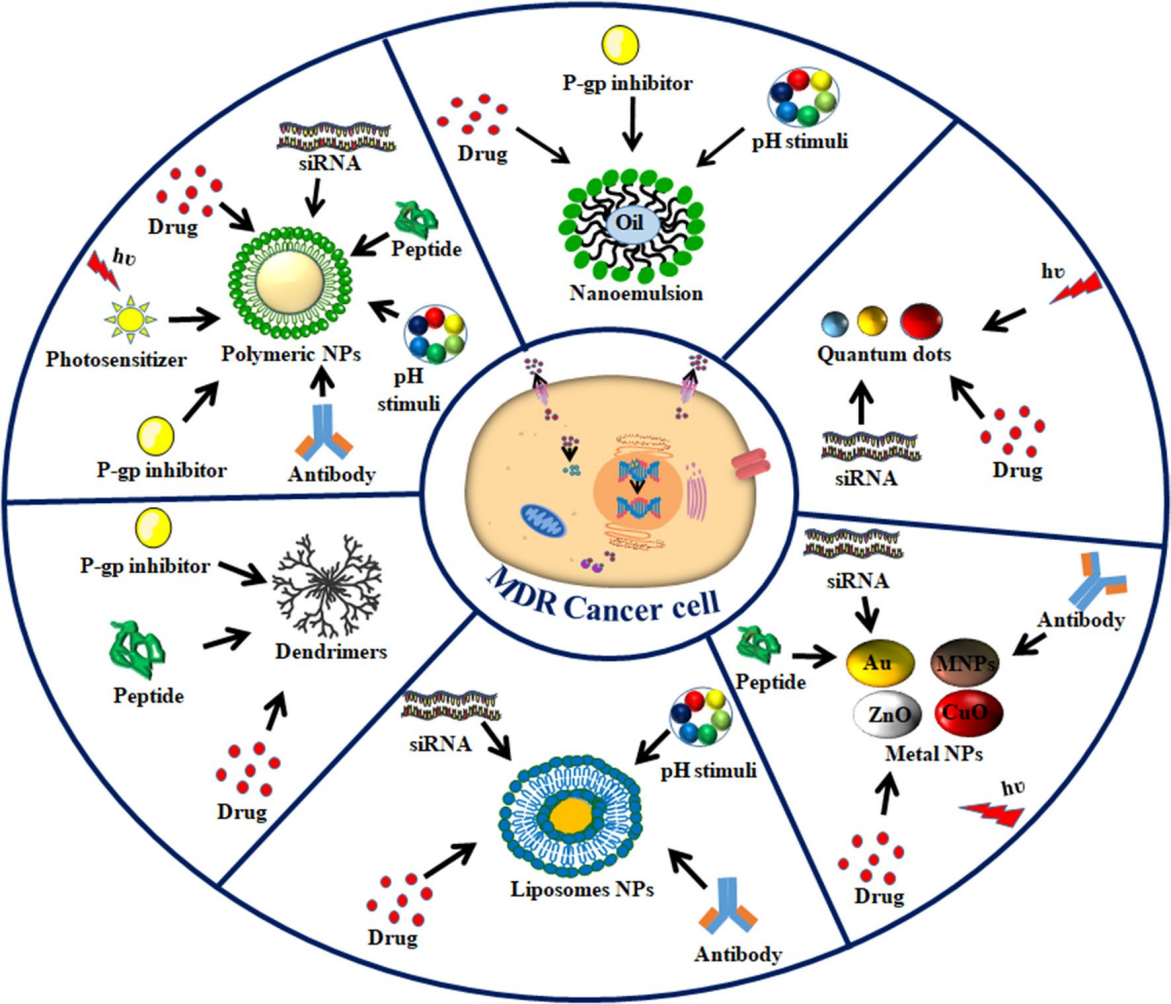
Different nanoparticles designed to overcome cancer MDR. Organic, lipid, polymer, metallic and quantum dots-based nanomaterials decorated with ligands for the co-delivery of chemotherapeutic drugs and siRNA to overcome cancer MDR in resistant cells. The drugs are released in the cancer cells in response to external stimuli, resulting in the inhibition of ABC drug efflux pumps, thereby sensitizing multidrug-resistant cells

Polymeric nanomaterials have been found to play a crucial role in the delivery of dual chemotherapeutic drugs for the reversal of MDR. In fact, a polymeric liposome was the first nanoformulation approved by FDA to be used as a nanotechnology-based anticancer therapeutic [75]. These nanoparticles are colloidal, biocompatible and biodegradable nanomaterials that entrap or encapsulate hydrophobic drugs such as cyclosporin, curcumin, paclitaxel and oxaliplatin in their matrices to improve their bioavailability in cells [96]. Table 2 lists the various types of polymeric nanomaterials that have been investigated for the reversal of cancer MDR. These nanomaterials are highly stable and have the intrinsic property of sustained and controlled drug release as compared to liposomes and micelles. Natural biopolymers such as chitosan, sodium alginate as well as some other synthetic polymers including hydroxypropyl methylcellulose (HPMC), Poly (lactic-co-glycolic acid) (PLGA), Poly-l-lysine (PLL), and *N*-(2-hydroxypropyl)-methacrylamide (HPMA) are commonly used for nanoformulation synthesis and drug delivery [97]. Polymeric nanoparticles provide sustained release of drugs, prevent drug metabolism and detoxification and have a long circulation time, avoiding clearance from the system and enhancing uptake within cells [98]. Many polymeric nanoparticles loaded with chemotherapeutic drugs and P-gp inhibitors have been studied to modulate ABC efflux transporters and enhance the intracellular accumulation of anticancer drugs in MDR tumor cells [99–101]. Le and co-workers, for example, evaluated doxorubicin-loaded liponanoparticles (LNPs) in order to bypass the P-gp efflux mechanism in doxorubicin-resistant MCF-7/ADR breast cancer cells. The drug-loaded polymeric nanoparticles significantly increased the accumulation of doxorubicin in the nuclei of drug-resistant cells [102]. In another study, curcumin and nutlin-3a in PLGA functionalized with folate reversed MDR through downregulation of *MRP1* via inhibition of NF-κB in retinoblastoma Y79 cells [103]. Figure 4 shows different types of nanomaterials such as organic polymer, lipid, metallic and quantum dost-based nanomaterials functionalized with various ligand molecules for the co-delivery of chemotherapeutic drugs to overcome cancer MDR in resistant cells.

**Table 2:** Polymeric and liposomal nanomaterials used to reverse cancer MDR

S. No	Nanoparticles (NPs)	Chemotherapeutic drugs	Experimental model	Mechanism of action	Refs
1	Tween 80 and PEG coated PBCA NPs	Doxorubicin	Human colon (SW620 and SW620/Ad300) and NSCLC (NCI-H460 and NCI-H460/MX20)	PEG and Tween 80 act as P-gp inhibitors and block the doxorubicin efflux from the cells and doxorubicin mediated toxicity	[261]
2	PLGA NPs	Doxorubicin and Verapamil	Human breast cancer (MCF-7 cells)	Inhibition of P-gp efflux pump and enhanced accumulation of drugs and cytotoxicity	[262]
3	Nano PEG-ADDC	Irinotecan and YC-1 (3-(5'-hydroxymethyl-2'-furyl)-1-benzylindazole)	Non small cell lung cancer (A549 cells)	Downregulation of HIF-1α and VEGF proteins also enhanced intracellular drug retention	[263]
4	Folate-biotin conjugated starch NPs	siRNA-IGF1R and Doxorubicin	Human lung carcinoma (A549 cells)	Downregulation of IGF1R protein expression and drug uptake and cytotoxicity	[264]
5	HPMA-gelatin NPs	Doxorubicin and Glycyrrhetinic acid (GA)	Human hepatoma (HepG2 and HepG2/ADR cells) and BLAB/c nude mice	P-gp efflux pump downregulation by GA, enhanced mitochondrial ROS generation, higher uptake of drugs in cells and suppression of tumor growth	[265]
6	Folate modified DSPE-MPEG-PLGA NPs	Phephobide a (Pba)	Human gastric (MKN 28 cells) and nude mice	Bypass the P-gp efflux pump and elevated levels of intracellular drug accumulation	[266]
7	Hyaluronic acid conjugated PLGA NPs	Paclitaxel and FAK specific siRNA	Human ovarian cancer (A2780, A2780-CP20 cells, SKOV3, SKOV3-TR, HeyA8 and HeyA8-MDR cells) and PDX mouse model	FAK gene silencing, enhanced accumulation of drug, bypassing the P-gp efflux pumps and suppression of tumor growth and development in PDX models	[81]
8	PEG-PLA NPs	Cyclosporin A and Gefitinib	Human non-small cell lung cancer (PC-9 and PC-9-GR cells, H1975 cells) and BALB/c mice PC-9-GR and H1975 xenografts	Inhibit EGFR tyrosine kinase disturb the downstream STAT3/Bcl-2 signaling transduction of leads to inhibition of cancer cell progression	[153]
9	TPGS-PLGA NPs	Doxorubicin and Metformin	Human breast cancer (MCF-7/DOX cells)	Inhibit P-gp to reduce drug efflux and enhance intracellular doxorubicin accumulation and reduce the cellular ATP content	[147]
10	Mannosylated albumin NPs	Disulfiram/Cu complex and regorafenib	Human cells (HCT8/ADR and HUVEC cells) mouse macrophage cells (RAW 264.7 and L929 cells)	Chemosesitization through ROS generation and enhanced apoptotic cell death	[267]
11	FA-PEI-PEG conjugated nanographene	P-gp specific siRNA (siP-gp) and Doxorubicin	Human breast cancer (MCF-7 and MCF-7/ADR cells)	siRNA-mediated P-gp gene silencing, enhanced doxorubicin retention and toxicity	[268]
12	Amphiphilic poly-Jug-DA-b-PEG NPs	P-gp specific siRNA (siP-gp) and Doxorubicin, Juglanin	Human lung cancer (A549 and H69 cell lines) and nude BLAB/c mice	Inhibition of P-gp gene silencing, drug uptake, inhibiting tumor growth	[269]
13	Anisamide-PLGA NPs	Resveratrol and Doxorubicin	Human breast cancer cell lines (MCF-7/ADR, MDA-MB-231/ADR cells) and BALB/c nude mice	Inhibition of the expression of MDR-linked transporters P-gp, MRP-1, BCRP and downregulation of NF-κB to enhance apoptosis	[270]
14	Chitosan modified TPGS-b-(PCL-ran-PGA) NPs	siHIF and Cisplatin	Human nasopharyngeal cancer cell line (CNE-2 cells)	MDR1/P-gp gene silencing via siHIF gene and enhanced sensitivity of cisplatin to cells	[175]
15	PLGA-TPGS NPs	Docetaxel and Poloxamer 235	Human breast cancer (MCF-7/TXT cells) and SCID mice	P-gp inhibition and docetaxel accumulation and tumor suppression	[271]
16	Chitosan NPs	Gefitinib and shMDR1 gene	Gefitinib resistant HeLa cells	shMDR1-mediated anti-DNA enzyme degradation activity and inhibition of MDR1 gene expression	[272]
17	TPGS conjugated chitosan NPs	Doxorubicin	Human hepatocarcinoma (HepG2 and BEL-7402) and human breast cancer (MCF-7/DOX cells) and BEL-7402/5-Fu cells	NP-mediated P-gp efflux pump blocking and downregulation of cellular ATP levels	[273]
18	mPEG-b-PLA polymersomes	Bcl-xL siRNA and Doxorubicin	Human gastric cancer (MKN-45 and MKN-28 cell lines)	siRNA-mediated downregulation of Bcl-xL, enhanced intracellular drug retention and reversal of MDR	[274]
19	Chitosan-PBCA NPs	Curcumin and Doxorubicin	Human breast cancer MCF-7 and MCF-7/ADR cells	Downregulation of P-gp efflux pump, NF-κB and elevated level of drug retention and cytotoxicity	[275]
20	Folate-PLGA NPs	Nutlin-3a and Curcumin	Human retinoblastoma Y79 cells	Curcumin-mediated MRP-1 and LRP downregulation via modulation of NF-κB translocation	[246]
21	PLGA NPs	Doxorubicin	Human ovarian (SKOV-3) and uterine (MES-SA/Dx5) cells	Drug resistance is overcome via enhancing intracellular drug uptake and nuclear retention	[276]
22	PLGA NPs	Curcumin and Doxorubicin	Human leukemia (K562) cells	Curcumin-mediated P-gp inhibition, downregulation of MDR1, NF-κB and Bcl-2 gene expression	[277]
23	AOT-Sodium alginate	Methylene blue and Doxorubicin	Primary mammary adenocarcinoma cells (JC cells) and female BALB/c mice	Methylene blue acts as P-gp inhibitor, enhances the inhibition of tumor progression and increases apoptotic mechanisms	[278]
24	Dextran sulphate-PLGA hybrid NPs	Vincristine sulfate	Human breast cancer (MCF-7 and MCF-7/ADR)	Inhibition of P-gp efflux ability by blocking of efflux transporter by NPs and enhanced intracellular drug retention	[279]
25	NIPMAm based core–shell hydrogels	siRNA and Docetaxel	Human ovarian cancer cell lines (Hey and SKOV-3 cells)	Loss of EGFR expression due to EGFR gene silencing via siRNA for increased sensitivity of docetaxel	[280]
26	Folic acid conjugated hydroxypropyl chitosan NPs	Antisense oligodeoxynucleotides as ODNs	Human carcinoma dox resistant cells (KB-A-1 cells) and BALB/c nu/nu KB-A-1 xenograft mice	Inhibition of expression of MDR1 gene and P-gp efflux pumps	[281]
27	Biotin conjugated PLGA-PEI NPs	Paclitaxel and P-gp targeting siRNA	Primary mammary adenocarcinoma cells (JC cells) and female BALB/c mice	Enhanced intracellular retention of paclitaxel and silencing of MDR1 gene that encodes for P-gp efflux pump	[282]
28	Biotin-PLGA NPs	Tariquidar and Paclitaxel	Ovarian cancer cell lines NCI/ADR-RES cells and BLAB/c mice	Inhibition of P-gp pump-mediated drug efflux from cells, enhanced drug retention and cytotoxicity and tumor growth inhibition	[283]
29	PLGA NPs	Vincristine and Verapamil	Human Breast cancer cells (MCF-7/ADR cells)	P-gp inhibition via verapamil and elevated intracellular drug retention	[284]
30	PEO-modified PBAE NPs	MDR1 gene silencing siRNA and Paclitaxel	Ovarian cancer (SKOV and SKOV3_TR_) cells	Anti-MDR1 gene silencing via siRNA leads to drug retention and cytotoxicity in cells	[285]
31	PEO modified PLGA/PBEA blend NPs	Paclitaxel and Ceramide	Human breast cancer (MCF-7 and MCF-7_TR_ cells) and MCF-7/ADR nu/nu xenograft model	Enhanced intracellular accumulation and lower clearance rate of paclitaxel	[286]
32	PEO-PCL NPs	Paclitaxel and C6-ceramide	Ovarian cencer (SKOV3 and SKOV3_TR_) and nu/nu xenograft model	Drug combination enhances apoptosis and inhibits tumor progression	[287]
*Liposomal nanoformulations*					
33	TPGS-liposomes	Docetaxel and Coumarin-6	Human lung cancer (A549 and A549/DDP cells) and nude mice	Inhibition of P-gp pump activity preventing the efflux of drug from cells. Enhanced intracellular and antitumor activity of drugs	[107]
34	Protoporphyrin IX (PpIX) doped liposome	Doxorubicin	Human breast cancer (MCF-7/ADR cells) and nude mice	Photodynamic-mediated disruption of P-gp efflux pumps by PpIX with enhanced intranuclear drug accumulation and suppression of tumor growth	[108]
35	Amphiphilic cationic phospholipids	Cabzitaxel and silibinin	Prostate cancer (PC-3 and DU-145 cells)	Active targeting of CD44 cell markers to target CSCs and overcome MDR	[288]
36	PEG-PLL-DMA Liposome	NO donar (DETA NONOate) and Paclitaxel	Human lung cancer (A549/T cells) and nude mice	Enhanced drug release and accumulation and downregulation of the expression of P-gp efflux pump with suppression of tumor development	[289]
37	Liposome	Doxorubicin and Aptamer AS1411	Human breast cancer (MCF-7/ADR cells)	Enhanced nuclear uptake, release of drug in nuclei and bypassing P-gp efflux pumps	[290]
38	CL-R8_LP-SPC:CHO:CHO-PEG_2000_-R8:CHO-S–S-PEG_5000_	Doxorubicin and verapamil	Human breast cancer (MCF-7 and MCF-7/ADR cells)	Liposome-mediated P-gp bypassing for intracellular drug retention and enhanced cytotoxicity	[105]
39	Liposome (Lipodox)	Doxorubicin	Human colon cancer (HT29 and HT-29-dx cells)	Alteration of the P-gp raft composition with impaired P-gp transport function and ATPase activity	[76]
40	Liposome polycation DNA (LPD) NPs	siRNA (VGFR and c-myc) and Doxorubicin	Ovarian cancer cell lines (NCI/ADR-RES and OVCAR-8 cells) and NCI/ADR-RES xenograft female nude mice cells	siRNA-mediated silencing of MDR1 gene. Lipid NPs also act as P-gp inhibitors with elevated levels of intracellular Doxorubicin	[291]
41	Amphiphilic phospholipid and cholesterol with transferrin	Doxorubicin and verapamil	Leukaemia (K562 and K562/DOX) cells	Inhibition of the P-gp efflux pump	[292]
42	Anti-transferrin receptor monoclonal antibody (OX26 mAb) conjugated liposome	Digoxin and Propidium Iodide	Rat brain capillary endothelial cells (RBE4)	OX26 mAb and digoxin conjugated liposomes effectively bypass the P-gp efflux pump	[106]
43	Cardiolipin-phosphotidylcholine based liposomes	Doxorubicin	Human breast cancer (MCF-7, MCF-7/ADR) and ovarian cancer (SKOV3, SKVLB) cells	Liposomal formulation modulates the intracellular drug distribution and retention	[293]
44	Liposome	Doxorubicin	Human leukemia (HL-60/VCR, HL-60/ADR cells)	Inhibition of the P-gp drug efflux pump via direct interaction, with elevated intracellular drug retention	[294]
*Solid Lipid Nanoparticles (SLNs)*					
45	SLNs	Linagliptin	Human colorectal cancer (Caco-2 cells) and Albino Wistar rats	Inhibition of P-gp drug efflux and lymphatic targeting	[222]
46	SLNs	Curcumin	Human breast cancer (MCF-7, MDA-MB-231) cells and murine mammary (JC cells) and BALB/c mice	Inhibition of Akt/IKKα-β/NF-κB signaling and inhibition in transcriptional activation of P-gp promoter p65/p50 NF-κB	[295]
47	SLNs	Doxorubicin and Quinazolinone derivative (QZO-DER)	Human colorectal cancer (HCT-116 cells), human lung carcinoma (A549 cells) and human breast cancer (MCF-7 and MDA-231 cells)	Enhanced cellular uptake of SLNs conjugated with drugs and associated cytotoxicity to cell lines	[296]
48	SLNs	Paclitaxel	Human breast cancer (MCF-7, MCF-7/ADR cells)	Intracellular uptake of drug and evasion of the P-gp efflux pumps	[297]
50	Nanolipid carriers (NLCs)	β-lapachone and Doxorubicin	Human breast cancer (MCF-7/ADR cells) and BLAB/c nude mice	Lapachone inhibits P-gp expression, diminishes ATP levels and downregulates HIF-1α and NF-κB expression	[298]
51	TPGS-Brij78 coated SLNs	Curcumin and Piperine	Human ovarian resistant cancer (A2780/Taxol cells)	TPGS and Brij78-mediated P-gp inhibition with enhanced drug retention and elevated cytotoxicity	[299]
52	SLNs	Paclitaxel and Curcumin	Human breast cancer (MCF-7/ADR) cells	Curcumin inhibits P-gp expression and enhances intracellular drug retention and cytotoxicity	[300]
53	Nanolipid carriers (NLCs)	Doxorubicin and Vincristine	B-lymphoma (LY-I cells) and BALB/c mice	Enhanced intracellular uptake, bypassing the P-gp efflux pump and improved suppression of tumors	[301]
54	Cholesterol-PEG coated SLNs	Doxorubicin	Human breast cancer (MCF-7, MCF-7/ADR cells) and xenograft nude mice	Inhibition of P-gp drug efflux activity, enhanced drug retention and cytotoxicity	[302]

### Liposomal nanoformulations

Liposomal nanoformulations are spherical vesicles that encompass amphiphilic phospholipids and cholesterol associated with an aqueous lumen. Liposomes can allow the encapsulation of both hydrophobic as well as hydrophilic chemotherapeutic drugs within their cores. A liposomal nanoformulation was the first clinically approved nanosystem for anticancer drug delivery [104]. Table 2 provides a comprehensive list of various liposomal and solid lipid nanoformulations used for the reversal of MDR. Liposomes can also be utilized as a co-delivery system to deliver a chemotherapeutic agent along with inhibitors to sensitize cancer cells to anticancer drugs [105]. In one study, the co-encapsulation of doxorubicin and verapamil in liposomal-mediated delivery was found to overcome P-gp-mediated MDR in human breast cancer cells with reduced toxicity in vital non-target organs [105]. Tang and co-authors synthesized a liposomal formulation and decorated its surface with octa-arginine (R8), which acts as a cargo peptide and delivers the liposomal formulation into cells. This nanoformulation was revealed to cause significant inhibition of tumor growth in female nude mice with negligible distribution in healthy tissues and organs. Thus, liposomal nanoformulations offer a platform for co-administration of chemotherapeutic drugs in combination with inhibitors of ABC drug transporters to eliminate MDR in both cellular and animal models [105–108]. Several liposomal nanoformulations of chemotherapeutic drugs are under clinical studies and approved by the FDA for the treatment of different subtypes of cancers [109, 110]. These liposomal nanoformulations can deliver the drugs for maximal synergy at a specific molar ratio suitable for the tumor microenvironment. For example, the FDA-approved nanoliposome Vyxeos was used for the co-delivery of cytarabine and daunorubicin to achieve effective treatment of acute myeloid leukemia (AML) [111].

### Micellar nanoparticles

Micelles are specialized nanomaterials obtained by self-assembly of hydrophilic and hydrophobic blocks in an aqueous environment with a hydrophobic core. The hydrophobic core has the advantage of being able to entrap hydrophobic drugs within its core. Polymers such as poly (aspartic acid) (PAA), poly (caprolactone) (PCL), poly (lactic-co-glycolide) (PLG) and polyethylene glycol (PEG) are used for micelle formation. Table 3 shows recent developments in the application of various nanomicelles to overcome cancer MDR. Several polymeric micelles loaded with certain chemotherapeutic agents (doxorubicin, cisplatin and paclitaxel) have been evaluated for their anticancer efficacy in experimental as well as clinical studies. Lv et al. demonstrated the use of polymeric micelles (PEG_2k_-PLA_5k_) for co-delivery of doxorubicin with curcumin to reverse MDR via dual-drug based nanomicelles in drug-resistant MCF-7/ADR cells and in a xenograft model [112]. Nanomicelles were also used for co-delivery of P-gp-specific siRNA and anticancer drugs in a single system for synergistic and effective anticancer therapy. For example, Zhang et al*.* applied a triblock polymer (NSC-PLL-PA) for co-delivery of si-*MDR1* RNA and doxorubicin to resistant HepG2/ADM cells and a xenograft model. Moreover, nanomicelles were observed to accumulate in tumors 24 h post-injection and inhibit tumor growth [113]. Various polymeric nanomicelles have been found to be effective and have achieved success in different clinical stages. Genexol-PM, nanomicelles loaded with paclitaxel, has been approved by the FDA for use in patients to treat breast cancer. Preclinical in-vivo studies revealed a threefold increase in the maximum tolerated dose of paclitaxel and enhanced antitumor activity as compared to the free drug. Another advantage of using nanomicelles is their hydrophilic outer shells. Such micellar nanomaterials have prolonged circulation time and accumulate in tumor tissues via the EPR mechanism [114].

**Table 3:** Nanomicelles and nanoemulsions used to overcome cancer MDR

S. No	Nanoparticles (NPs)	Chemotherapeutic drugs	Experimental model	Mechanism of action	Refs
*Nanomicelles*					
1	TAT-TPGS-PEG-b-P(ALA) nanomicelles	Doxorubicin	Human breast cancer (MCF-7 and MCF-7/ADR cells)	Inhibition of P-gp efflux pump and intracellular drug retention and enhanced cytotoxicity	[303]
2	Folic acid conjugated P(OEGMA300)-st-P(HEMA-g-LA) nanomicelles	Doxorubicin	Human cervical cancer (HeLa cells)	Enhanced intracellular drug accumulation and tumor microenvironment-triggered drug release and cytotoxicity	[304]
3	Polycarbonate based NO nanomicelles	Doxorubicin	Human breast cancer (MCF-7/DOX^R^ cells)	Nitric oxide-mediated P-gp inhibition, intracellular drug accumulation and cytotoxicity	[305]
4	Nitrate functionalized TPGS nanomicelles	Doxorubicin, adjudin and nitric oxide	Human breast cancer (MCF-7/ADR cells), murine mammary carcinoma (4T1 cells) and BALB/c mice	Controlled drug release, enhanced cytotoxicity, P-gp inhibition, reduction in tumor growth and reversal of MDR	[306]
5	Poly (β-amino ester) polymer-based nanomicells	Doxorubicin	Human breast cancer (MCF-7 and MCF-7/ADR cells)	Inhibition of P-gp efflux pump by polymer, lysosomal escape and intracellular drug retention	[307]
6	FA-Chitosan coated nanomicelles	siRNA and Doxorubicin	Murine breast cancer (4T1, 4T1/MDR cells) and 4T1/MDR xenograft mice	Elevated intracellular drug accumulation and downregulation of MDR1 gene for the suppression of tumor growth	[308]
7	Thermosensitive Hydrogel	Doxorubicin and Docetaxel	Murine CT-26 cell line and CT-26 xenograft mice model	Sustained drug release and enhanced retention and antitumor activity with minimized inherent side effects in mice	[309]
8	Lysine linked d-α-TPGS2000 succinate (PLV_2K_) nanomicelles	Doxorubicin	Human colorectal adenocarcinoma cell line (Caco-2 cells)	Elevated intracellular accumulation of doxorubicin by uncompetitive P-gp ATP base inhibition	[310]
9	Polyvinyl caprolactam-polyvinyl acetate-polyethylene micelles (Soluplus®)	Doxorubicin	Human breast cancer (MCF-7 and MCF-7/DOX cells) and nude BALB/c mice	Inhibition pf P-gp drug efflux and alteration in membrane fluidity further enhance the intracellular drug retention and associated toxicity with antitumor activity	[311]
10	PEG-PE/VitaminE nanoemulsion	Paclitaxel and curcumin	Human ovarian cancer cell lines (SKOV3 and SKOV3_TR_ cells) and female nude mice	Bioavailable curcumin inhibits the NF-κB and also acts as an inhibitor for P-gp efflux transporters and elevated intracellular paclitaxel	[146]
11	PCL-PEG nanomicelles	Doxorubicin and siHIF	Prostate cancer (PC-3) cells and xenograft mice	siHIF-mediated MDR1 gene silencing, disruption of angiogenesis and enhanced intracellular doxorubicin levels	[174]
12	Pluronic (P-105) based nanomicelles	Doxorubicin and Ruboxyl	Human leukemia cell line (HL-60 cells), Human ovarian cancer cell lines (A2780S and A2780/ADR cells)	Surfactant-based nanomicelles facilitate the delivery of drugs to resistant cells and also enhance their intracellular retention	[312]
*Nanoemulsion*					
13	Nanoemulsion	Paeonol	Human colorectal adenocarcinoma (Caco-2 cell lines) and male SD rats	Nanoemulsions bypass P-gp efflux pumps and suppress tumor growth	[313]
14	Folate conjugated nanoemulsion	Docetaxel	Human epithelial cell lines (KB-WT and KB-PR10 cells) and C57BL/6 J transgenic mouse	Bypasses efflux pumps and downregulates P-gp efflux with enhanced antitumor activity	[314]
15	Amphiphilic dendrimer	Doxorubicin	Human breast Cancer (MCF-7S, MCF-7R cells), Human prostate cancer (PC-3 cells), Human hepatocarcinoma (HepG2 cells), Human cervical cancer (HeLa cells) and Mice models (NSG mice and C57BL/6 mice)	EPR-mediated efficient doxorubicin delivery prevents systemic toxicity and overcomes drug resistance	[315]
16	Capryol 90- nanoemulsion	Paclitaxel	Human colorectal cancer (Caco-2 cells)	Inhibit P-gp efflux and enhance drug retention and associated toxicity	[316]
17	Flaxseed oil-DSPE-PEG2000) nanoemulsion	Curcumin and Paclitaxel	Ovarian cancer (SKOV and SKOV3_TR_) cells	Inhibit NF-κB activity and downregulate P-gp expression with elevated intracellular drug retention	[317]
18	Polyalkylcyanoacrylate NPs	Cyclosporin A and Doxorubicin	Mouse leukemia (P388/ADR cells)	Inhibition of P-gp efflux pump by cyclosporine A with enhanced doxorubicin retention and associated cytotoxicity	[318]

### Nanoemulsions

Nanoemulsions (oil/water) are biocompatible, highly stable nano-size (10–1000 nm) emulsions that are frequently used to entrap and improve the delivery of hydrophobic drugs and pharmaceutically active compounds [115]. Table 3 shows various nanomicelles and nanoemulsions that have been used to overcome cancer MDR. Through nanoemulsion, the co-administration of a different combination of chemotherapeutic drugs and/or efflux transporter modulators can efficiently be introduced into cancer cells. These nanoemulsions play a significant role to overcome MDR [116]. Albumin-bound nanoparticles (nab™) have been widely used for tumor treatment due to elevated albumin accumulation within tumors. The nab-paclitaxel nanoformulation (Abraxane®) was given FDA approval for the treatment of metastatic breast cancer and non-small cell lung cancer [117]. Co-delivery of docetaxel and thymoquinone in borage oil-based nanoemulsion reduces the concentration necessary for effective treatment in breast cancer (MCF-7 and MDA-MB-231) cells as compared to drug-free treatment [118].

### Dendrimers

Dendrimers are nano-size hyper-branched, spherical polymeric nanomaterials with symmetric core and end groups that facilitate surface conjugation and modification. Anbazhagan et al*.,* employed polyamidoamine (PAMAM) dendrimers for the co-delivery of ferulic acid and paclitaxel. These dual-drug loaded PAMAM dendrimers were also decorated with arginyl-glycyl-aspartic acid (RGD) to combat MDR mediated by P-gp in drug-resistant KB Ch^R^-8–5 cells. These results revealed the enhanced intracellular accumulation of paclitaxel in cells and also indicated increased pro-apoptotic protein expressions of caspase 3, caspase 9, p53 and Bax [119]. Similarly, Liu et al*.* demonstrated the role of dual-functionalized PAMAM dendrimers in the inhibition of P-gp function in Caco-2 and MDCK/MDR1 cells [120].

### Metallic nanoparticles

Several metals and metal oxides have attracted intense biomedical attention for their use as nanomaterials in diagnosis, drug delivery and therapy. Gold (Au) and iron oxide nanoparticles (Fe_3_O_4_ NPs) have intrinsic properties that make them ideal nanosystems to facilitate therapies using radiation, photodynamics and hyperthermia. Iron oxide nanoparticles can be utilized as a contrast agent to improve conventional MRI imaging [121]. Various metallic nanomaterials used for the reversal of drug resistance are listed in Table 4.

**Table 4:** Application of metallic nanomaterials to overcome cancer MDR

S. No	Nanoparticles (NPs)	Chemotherapeutic drugs	Experimental model	Mechanism of action	Ref
1	Ferric-tanic acid nanocapsule	Doxorubicin and Glucose oxidase (GOx)	Human liver (HL-7702 cells), murine breast cancer (4T1 cells) and BALB/c mice	ATP downregulation triggers the suppression of P-gp efflux and enhanced intracellular drug uptake and retention and suppression of tumor development	[319]
2	PHB coated magnetic NPs	Etoposide and MRP-1 specific siRNA	Human breast cancer (MCF-7/S and MCF-7/1000ETO cells)	*MDR1* gene silencing via siRNA and inhibition of drug efflux and higher drug uptake and cytotoxicity	[320]
3	Calcium phosphate nanoparticles (TCaNG)	Doxorubicin	Human breast cancer (MCF-7/ADR cells) and nude mice	Disruption of calcium ion-mediated mitochondrial homeostasis, blocking ATP activity and inhibiting the biosynthesis and function of P-gp efflux transporters in cells	[155]
4	Sulfhydryl functionalized-Fe_3_O_4_@polydopamine-mesoporous silica NPs	Doxorubicin	Human hepatocellular carcinoma (HepG2 cells)	Reversal of MDR via NP-mediated photothermal effect and efficient inhibition of tumor development	[321]
5	PEO-AgNPs loaded nanofiber	Niclosamide	Human lung cancer (A549 cells) and human breast cancer (MCF-7 cells)	Enhancement of pro-apoptotic genes, ROS-mediated cell death due to elevated accumulation of drugs in cells	[322]
6	AgNPs	Doxorubicin	Human breast cancer (MCF-7 and MCF-7/KCR cells)	AgNP-mediated enhanced ROS generation, mitochondrial damage and inhibition of P-gp efflux pumps	[323]
7	AuNRs	Doxorubicin and Polycurcumin	Human breast cancer (MCF-7/ADR cells)	Nanorod-mediated photothermal effect, enhanced drug retention and cytotoxicity	[324]
8	SPION NPs	Conjugated linoleic acid	Murine breast cancer (4T1 cells)	PARPγ-mediated cytotoxicity and enhanced inhibition of P-gp efflux pumps	[325]
9	Hydroxyapatite-*β*-CD coated magnetic nanocomposite	Doxorubicin and Curcumin	Human breast cancer (MCF-7 and MCF-7/ADR cells) and BLAB/c mice	Curcumin-mediated inhibition of P-gp efflux, enhanced delivery and accumulation of drug via nanoclusters and suppression of tumor growth and development	[326]
10	Mucin-1 conjugated AuNRs	Doxorubicin	Human breast cancer (MCF-7/ADR cells)	Downregulation of P-gp efflux pumps and enhanced intracellular drug retention	[327]
11	AuNPs	Sorafenib	Human hepatocellular cancer (HepG2 resistant cells)	Reversal of drug resistance via targeting molecular machinery CD147, TGF-β and downregulation of ABCG-2 drug efflux pump	[328]
12	TAT-conjugated AuNPs	2-(9-anthracenylmethylene)-hydrazinecarbothioamide (ANS)	Human hepatocellular carcinoma (HepG2 cells) and human breast cancer (MCF-7 and MCF-7/ADR cells)	Bypassing P-gp efflux, enhanced delivery of drug to cells	[329]
13	*β*-CD coated AuNPs	Paclitaxel	Human lung cancer (H460 and H460_PTX_ cells)	Nanoconjugates evade P-g-mediated efflux and elevate intracellular drug levels	[330]
14	CuO and ZnO NPs	Vinblastin	Sea urchin embryos	Potential inhibitors of ABC efflux transporters	[131]
15	PLGA coated AuNPs	DR-4 and Doxorubicin	Human colon cancer (DLD-1 and DLD-1/DOX cells) and nude BALB/c mice	Enhanced chemo-photothermal therapy and cytotoxicity in cells and suppression of tumor growth	[79]
16	Lectin conjugated Fe_3_O_4_ NPs	Paclitaxel	Bcr-Abl positive cell lines	Instability of Bcr-Abl through JNK pathway activation and commencement of extrinsic apoptotic pathways	[130]
17	Nanodiamonds	Doxorubicin	Hepatoblastoma tumor modal (LT2-Myc cells and LT2- myc mice)	Overcoming transporter-mediated drug efflux and inhibition of cancer progression	[154]
18	OA coated-(Fe_3_O_4_) NPs	Daunorubicin and 5-bromotetrandrin	Human leukemia cell line (K562/A02 cells)	*MDR-1* gene downregulation and retention of daunorubicin	[80]
19	Fe_3_O_4_ NPs	MDR short hairpin RNA (shRNA)	Human leukemic cell line (K562/A02 cells)	Synergistic effect of Fe_3_O_4_ NPs and PGY1–2 (shRNA expression vehicle) to reverse MDR	[128]
20	Fe_3_O_4_ NPs	Cisplatin	Human ovarian cancer cells (SKOV3/DDP resistance cells)	Inhibition of P-gp-mediated efflux by downregulating the expression of BCL-2 and expression of P-gp gene and increasing cisplatin accumulation in cells	[129]

Green synthesized metal nanomaterials have attracted enormous attention and have been exploited for their biomedical applications. These green synthesized nanomaterials are prepared by using different plant parts, natural compounds, and microorganisms. Many reports have demonstrated the use of biosynthesized nanomaterials of different metals on cancer sub-types. Saravanan et al*.* in their systematic report elaborated comprehensive insights regarding the significant role of biogenic AuNPs in breast cancer treatment and molecular mechanisms for anticancer activity in in-vitro studies. The biogenic nanoparticles facilitate excessive production of ROS and apoptotic enzymes that contributes to higher cytotoxicity in cancer cells [122]. Mostafavi et al. described the efficiency of biogenic AgNPs and AuNPs for antineoplastic activity against leukemic models [123]. Barabadi et al*.* provided detailed information regarding the application of biologically synthesized AgNPs against lung cancer. Biogenic AgNPs were revealed to have elevated in-vitro anticancer efficacy, thereby facilitating the reversal of cancer MDR [124]. Another systematic review by Barabadi et al. demonstrated the relevance of biologically synthesized AuNPs for the diagnosis and treatment of lung, colorectal and cervical cancer cell lines using animal models [125–127].

Several reports indicate that metal nanomaterials are able to interfere with drug efflux transporters and cause the reversal of drug resistance by increasing drug retention and cellular bioavailability [80, 128–131]. Cheng et al*.* demonstrated the co-delivery of daunorubicin and 5-bromotetrandrin via magnetic nanoparticles (DNR/BrTet MNPs) to reverse P-gp-mediated MDR in K562/A02 leukemia cells. Their findings indicate that the transcriptional downregulation of the *MDR1* gene further aids in the reversal of MDR [80]. Noruzi et al*.* evaluated the effect of trimethoxusilylpropyl ethylenediamine triacetic acid (EDT)-coated and doxorubicin-conjugated iron oxide nanoparticles on human glioblastoma U251 cells and a mouse model for reversal of MDR. Their findings indicate that drug-conjugated magnetic nanoformulation activates multiple mechanisms to overcome drug resistance. It inhibited cell proliferation and enhanced apoptotic cell death. Furthermore, downregulation of the DNA repair gene and upregulation of caspase 3 and p53 genes were observed in U251 cells [132]. AuNPs have been found to contribute to the enhancement of chemotherapy and radiation in a size-dependent manner. Jiang et al*.* conjugated 2-(9-anthracenylmethylene)-hydrazinecarbothioamide (ANS) and 6-mercaptopurine (6-MP) with AuNPs and evaluated the resulting toxicity and drug resistance in MCF-7/ADR cells. Their findings indicated that smaller AuNPs have more efficient binding with P-gp, whereas larger-size nanoparticles avoid effective recognition by P-gp [133]. Rathinaraj et al*.* demonstrated the exploitation of folate-gold-bilirubin (FGB) nanoconjugates to overcome P-gp-mediated MDR in P-gp-overexpressing KB-Ch^R^-8–5 cells and in a xenograft mouse model. The results indicated the FGB nanoconjugate proved to be a potent inhibitor as compared to bilirubin and AuNPs alone. FGB nanoconjugates also induced intracellular ROS and initiated DNA strand breakage and other apoptotic changes in P-gp-overexpressing cells. The xenograft model treated with FGB nanoconjugates also revealed suppression of tumor growth with pronounced apoptosis [134]. Dearden et al*.* demonstrated that drug-functionalized gold nanorods (AuNRs) mediated P-gp trafficking in P-gp + J774.2 cells. Treatment with AuNRs containing azithromycin (Azith-AuNRs), clarithromycin (Clarith-AuNRs) and tricyclic ketolide (TriKeto-AuNRs) led to ligand-dependent accumulation and inhibition of the efflux of these nanorods by P-gp. Increased intracellular accumulation of AuNRs was observed for nanorods conjugated with P-gp substrates (Azith-AuNRs and Clarith-AuNRs), while nanorods conjugated with low-affinity P-gp substrates (TriKeto-AuNRs) was unaffected [135].

### Quantum dots

Quantum dots (QDs) are nanosized semiconductor particles with advantageous optical and electrical properties that have been successfully employed in several biomedical applications. QDs generate intracellular ROS, thereby causing cancer cell death through oxidative DNA damage [136]. Furthermore, QDs and carbon-based nanomaterials have been employed to conjugate drugs, antibodies and adjuvants to enhance anticancer therapeutic efficacy [137, 138]. In one study, P-gp-miR-34b and P-gp-miR-185 conjugated with CdSe/ZnS-MPA QDs and CdSe/ZnS-GSH QDs significantly inhibited P-gp expression in lung cancer A549 cells [139]. Graphene-based QDs (GQDs) have also been evaluated for their ability to modulate P-gp-mediated MDR. Single GQDs are able to downregulate multiple MDR-linked genes by interacting with their respective C-rich promoters. Furthermore, increased drug uptake and retention were observed along with suppression of MDR-related genes in MCF-7/ADR cells [140]. Table 5 lists published reports on the reversal of cancer MDR by QDs and carbon-based nanomaterials.

**Table 5:** Reversal of cancer multidrug resistance via quantum dots, carbon-based nanomaterials and mesoporous nanoparticles

S. No	Nanoparticles (NPs)	Chemotherapeutic drugs	Experimental model	Mechanism of action	Ref
*Quantum Dots (QDs)*					
1	Elacridar conjugated QDs	Doxorubicin	Human hepatocellular carcinoma (Bel-7402/ADR Bel-7402/ADR cells)	Specific binding of QDs to P-gp efflux pump active site, inhibition of drug efflux and enhanced doxorubicin retention	[331]
2	MPA-COOH- CdTe QDs	–	Human breast cancer (SK-BR-3 cells)	Small size QDs interact with ABC efflux transporters and block efflux efficacy	[332]
3	Cysteamine-CdTe QDs	Daunorubicin and Gambogic acid	Lymphoblastoid cells (Raji, Raji/DNR) and BALB/c nude mice	Downregulation of P-gp protein expression, drug retention and enhanced apoptotic mechanism	[333]
4	CdTe-QDs	Daunorubicin	Human hepatocarcimoma (HepG2/ADM) cells and nude mice	Inhibition of P-gp drug efflux pumps and overexpression of apoptosis-related caspase proteins with inhibition of tumor development	[334]
*Multiwalled Carbon Nanotubes (MWCNTs)*					
5	MWCNTs	N-Tamoxifen and Quercetin	Human breast cancer (MDA-MB-231 cells) and Wistar rats	N-TAM mediated P-gp inhibition and enhanced cellular uptake and cytotoxicity and control of tumor growth	[335]
6	P-gp antibody tagged-MWCNTs	–	Mouse fibroblast cells (T3T-MDR1 cells) and NCI/ADR-RES spheroids cells	P-gp specific cellular uptake of CNTs and enhanced phototoxicity in MDR cells	[306]
7	MWCNTs	–	Human hepatocarcinoma HepG2 cell line	Alteration of the mitochondrial membrane potential by elevated intracellular ROS, further inhibition of ABC-mediated efflux transporters	[336]
*Mesoporous nanoparticles*					
8	PEI-PEG functionalized mesoporous NPs	P-gp specific siRNA (siP-gp) and Doxorubicin	Human breast cancer (MCF-7/MDR cells) and MCF-7/MDR xenograft mice	P-gp silencing, intracellular drug uptake and retention and inhibition of tumor growth	[337]
9	Mesoporous silica NPs	γ-secretase inhibitors (GSIs)	Cervical cancer (HeLa cells), Human embryonic kidney (HEK293 cells), Breast cancer (T47D, MDA-MB-231, SK-BR-3, MDA-MB-468, MCF-7 cells) and MDA-MB-231 xenograft mice	MSN-GSI nanoformulations actively block Notch signaling in intestinal cancer stem cells	[213]
10	FITC-mesoporous silica NPs	Camptothecin	Pancreatic cancer (PANC-1, Capan-1, AsPc-1), Colon cancer (SW480) and stomach cancer (MKN45) cells	Enhanced drug delivery within cells and drug-mediated apoptotic cell death	[338]

### Mesoporous silica nanoparticles (MSNs)

Mesoporous silica nanoparticles are nanosize drug carriers that have gained attention as versatile drug delivery vehicles having a large surface area, high stability, negligible toxicity, customized pore size and ease of encapsulating various biogenic molecules. Table 5 lists mesoporous nanoparticles that have been used to overcome MDR. Liu and co-workers demonstrated the co-delivery of quercetin (a P-gp inhibitor) and paclitaxel in chondroitin sulphate-coated MSNs to reverse P-gp-mediated MDR. Their results indicated that increased drug release is dependent on the redox environment in MCF-7/ADR drug-resistant cells, ultimately resulting in downregulation of P-gp expression. In another report, higher intracellular drug retention, associated apoptosis and improved antitumor activity were observed in resistant cells and female nude BALB/c mice [141]. Also, Zhao et al*.* confirmed that pH-sensitive MSNs co-polymerized with d-α-tocopheryl polyethylene glycol 1000 succinate (TPGS) successfully deliver doxorubicin to resistant MCF-7/ADR cells. These MSNs showed clathrin-mediated endocytosis, and higher drug uptake and retention. The TPGS moiety of nanoformulation contributed exclusively to the inhibition of P-gp drug efflux in tumor-bearing SCID mice [142] and demonstrated the significant reversal of drug resistance.

## Recent advancements in nanotechnology to overcome MDR

Numerous engineered nanomaterials have emerged recently with the ability to deliver multiple agents such as chemotherapeutic drugs, adjuvants, and nucleic acids (DNA, siRNA, mRNA) to overcome MDR. These nanomaterials help to overcome MDR achieved by both efflux pump-mediated and efflux pump-independent mechanisms [101, 143].

### Nanomediated approaches to combat drug efflux pump-mediated MDR

P-gp transporters play a role in the development of clinical MDR in several cancer subtypes [144]. Therefore, inhibition of P-gp transport function has been considered an appropriate strategy to overcome MDR in cancer cells. Smart engineered nanomaterials have come to the rescue and have been shown to reverse efflux pump mediated-MDR by successfully altering the pharmacokinetic parameters that facilitate drug retention [145]. Several nonionic surfactants (Tween 80, vitamin E/TPGS, Brij 35, Pluronic, PEG and PEO, etc.) are now known to be able to inhibit P-gp activity [143]. These surfactants form strong hydrogen bonds with the transmembrane sequence of P-gp and engage the drug binding sites, enhancing drug absorption and retention. In one study, phosphatidylethanolamine (PE) conjugated with PEG (PEG-PE/vitamin E)-based nanomicelles were synthesized to co-encapsulate paclitaxel and curcumin for delivery to human ovarian adenocarcinoma SK-OV-3TR paclitaxel-resistant cells. The results of that study indicated successful delivery of drugs and inhibition of tumor growth in female nude mice by nanomicellar-mediated delivery [146]. Shafiei et al*.* conjugated TPGS-PLGA with doxorubicin and metformin for the co-delivery of P-gp inhibitor and chemotherapeutic drugs to inhibit drug efflux [147]. Drug delivery by polymer lipid nanoparticles (PLNs) has been shown to enhance chemotherapeutic efficacy and retention in resistant cells. Wong et al*.* conjugated doxorubicin with PLNs and evaluated their potential for drug delivery and P-gp inhibition in MDA435/LCC6/MDR1 and EMT6/AR1 resistant cell lines. The nanoformulations were able to bypass the efflux pumps as they were phagocytized into cells, thereby enhancing doxorubicin accumulation and retention in resistant cells as compared to free doxorubicin [148]. Joshi et al*.* demonstrated the reversal of hypoxia-mediated drug resistance in resistant A2780/ADR and MCF-7/ADR cell lines as well as in 3D spheroid cultures via co-delivery of doxorubicin and anti-P-gp siRNA (siP-gp)-conjugated PEGylated nanoparticles. The siRNA inhibits *MRP1* gene expression under hypoxic conditions, thereby increasing doxorubicin delivery to MDR cells [149]. Lamprecht et al*.* demonstrated the role of etoposide-conjugated lipid nanocapsules in the reversal of P-gp-mediated MDR in C6, F98 and 9L glioma cells. Etoposide intracellular efficiency was enhanced by lipid nanocapsules in P-gp-overexpressing MDR cells [150]. Some studies have been conducted to reverse MDR1 membrane pump-mediated cancer MDR with the employment of thermosensitive polymeric nanomaterials. Interestingly, Fan et al. developed beta cyclodextrin (β-CD)-based temperature-sensitive supramolecular nanoparticles by utilizing PEG-PNIPAAm for delivery of paclitaxel (β-CD-*g*-(PEG-v-PNIPAAm)_7_/PTX) and doxorubicin (β-CD-*g*-(PEG-v-PNIPAAm)_7_/Dox) both in-vitro (HepG2/MDR1 and H460/MDR1 cells) and in-vivo (HepG2/MDR1 bearing xenograft BALB/c mice). These novel nanoparticles facilitate the reversal of cancer MDR by enhancing the cellular uptake of nanoparticulated drugs, intracellular drug retention, and inhibiting pump-mediated drug resistance [151]. Cheng and co-workers developed a novel star-like thermoresponsive nanocarrier by using β-CD grafted with a copolymer of PNIPAAm-b-POEGA to form an inclusion complex for the delivery of doxorubicin and paclitaxel in HepG2/MDR1 and H460/MDR1 cells. Nanocarriers (β-CD-g-(PNIPAAm-b-POEGA)x/PTX@NPs) were highly stable and demonstrated enhanced cellular uptake of chemotherapeutic drugs. The nanocarriers were used at 37 °C (normal body temperature), thereby inhibiting MDR1-mediated cancer drug resistance. The β-CD-g-(PNIPAAm-b-POEGA)x/PTX@NPs) were shown to have an improved therapeutic effect attributable to enhanced cellular uptake and partial destruction of MDR1 membrane pumps with PEGylated nanocarriers in an in-vivo HepG2/MDR1 tumor xenograft nude mouse model [152]. Han et al*.* described the application of a PEGylated PLA nanosystem for the combined delivery of cyclosporine A and gefitinib in in-vitro and in-vivo cancer resistance models. Their findings indicated that the nanosystem disrupts EGFR-mediated downstream signaling cascades and eventually inhibits tumor growth and invasion. It also inactivates the function of the signal transducer and the activator of transcription-3 (STAT-3)-mediated signaling [153].

Nanodiamonds are carbon nanoparticles that offer binding sites for certain therapeutic agents. Reversible binding allows sustained release of drug at the target site, thereby achieving excellent biocompatibility. Chow et al*.* showed that nanodiamonds are an ideal drug delivery system that offers biocompatibility, drug conjugation, controlled release and enhanced aqueous dispersion properties. Nanodiamonds alter the tumor efflux pumps, hence facilitating doxorubicin intracellular retention and pronounced apoptosis in various human and murine breast cancer resistant cells and in a xenograft model [154].

Calcium phosphate-based nanomaterials are also used for the reversal of P-gp-mediated drug resistance via energy-dependent inhibition of efflux transporters [155]. Calcium phosphate nanoparticles loaded with doxorubicin decorated with an RGD peptide were evaluated for targeting MDR cells for reversal of P-gp-mediated drug resistance by inducing intracellular calcium ion bursting and designated as tumor Targeting Calcium ion NanoGenerator (TCaNG). The mechanism of action of this nanosystem was an initial burst of Ca^2+^ ions within mitochondria, which curbs cellular respiration by disturbing mitochondrial calcium ion homeostasis, blocking ATP production and further inhibiting P-gp-mediated cell resistance. Hypoxia conditions generated within cells due to suppressed cellular respiration also downregulate the hypoxia-inducible factor-1 alpha (HIF-1α) gene and inhibit expression of the P-gp efflux transporter. The study revealed that the TCaNG nanosystem inhibits the biosynthesis as well as functional activity of P-gp transporters and facilitates the reversal of tumor drug resistance in MCF-7/ADR resistant cells and nude mice [155]. These multi-targeted nanomaterials could be advantageous in preclinical and clinical applications.

Certain natural compounds have also been used to inhibit drug efflux transporters. Zhao et al*.* demonstrated the co-delivery of curcumin and paclitaxel via core–shell polymeric NPs in human ovarian cancer SKOV3 and SKOV3-TR30 cells and in tumor-bearing xenograft mice to reverse drug resistance. Their results demonstrated that the NPs are internalized via CD44 receptors present on the surface of ovarian cells. Curcumin was found to efficiently inhibit the P-gp drug efflux transporter, resulting in elevated intracellular paclitaxel retention, inhibition of cellular migration and cytotoxicity and enhanced reduction in tumor growth in a murine model [156].

Single-walled carbon nanotubes (SWCNTs) have been reported to efficiently overcome drug resistance in some experimental models. Li et al*.* demonstrated the co-delivery of anti-Pgp antibody and doxorubicin in SWCNTs in efforts to target and eliminate K562R leukemia stem cells. Their results clearly showed inhibition of tumor development and metastases [157]. In an earlier study, Li et al*.* conjugated both ABCG2 and ABCB1 sequences onto pH-sensitive carbonate apatite nanoparticles for dual siRNA-mediated targeting of human breast cancer cell lines (MCF-7). This dual targeting approach sensitized the MCF-7 cells and enhanced toxicity by more than 50% when treated with cisplatin, paclitaxel and doxorubicin. While single siRNA targeting resensitized the cells, the dual siRNA targeting approach offered enhanced toxicity [158].

### Nanomediated approaches to combat MDR not dependent on efflux pumps

Normal cells employ various repair mechanisms to avoid the replication of mutated DNA and to circumvent malignant transformation. If the damaged DNA is not repaired, the mutated cells are normally eliminated by apoptosis [159]. MDR not dependent on efflux pumps can also develop in cancer cells via activation of anti-apoptotic cellular mechanisms including elevated expression of the B-cell lymphoma 2 (Bcl-2) gene and inhibition of pro-apoptotic signals, or via HIF-1α and NF-κB [160–163]. NF-κB is responsible for the transcriptional regulation of several genes involved in cell proliferation, migration, invasion, apoptosis escape processes and survival. Atypical regulation of NF-κB has been shown to be crucial for the development of MDR.

### Nanomaterial-based approaches to combat tumor microenvironment-mediated MDR

The tumor microenvironment also plays an important role in MDR as well as cancer progression and development. Cancer cells and stromal cells embedded in the extracellular matrix play a crucial role in cancer cell invasion, metastasis and drug sensitivity [164]. Cancer cells are known to utilize more aerobic glycolysis than oxidative phosphorylation due to high levels of glycolysis and poor transportation of metabolites from cells. Lactic acid accumulation makes the intracellular environment acidic by increasing proton concentrations. The significant difference in pH (acidic pH in the extracellular matrix and neutral to basic pH in the intracellular environment) also influences the effectiveness of chemotherapeutic agents by ionizing them, hindering their ability to cross cell membranes and reducing intracellular uptake via transporters, leading to MDR in cancer cells [165, 166]. The tumor microenvironment possesses inimitable characteristics, contributing actively to the development of MDR. Smart nanomaterials utilize the physiological characteristics of the tumor cells and respond according to the tumor microenvironment, thus offering more effective treatment than conventional chemotherapy. Smart engineered nanoparticles respond according to the cellular pH, for the release of chemotherapeutic drugs at the tumor site. Several pH-sensitive polymeric nanomaterials have been extensively studied in efforts to overcome acidic tumor microenvironment-mediated drug resistance. Bahadur et al*.* synthesized poly (2-)pyridine-2-yldisulfanyl)ethyl acrylate) (PDS) nanoparticles loaded with doxorubicin and decorated with a cRGD peptide and observed their stability and drug release in both acidic pH and redox potential conditions in colon cancer HCT-116 cells. Their results indicated that these nanoparticles are a promising nanotherapeutic system [167]. Huo et al*.* employed a nanomicelle system for the co-delivery of the P-gp inhibitors disulfiram and paclitaxel in PEG-b-PLL/DMA with l-lysine side chains in efforts to reverse drug resistance. The nanomicelles tend to reverse surface charges depending on cellular pH conditions. Usually, nanomicelles have negative surface charge densities in neutral plasma circulations (pH 7.4) but they switch to a positive charge in an acidic tumor environment (pH 6.5–6.7). These positive surface charges facilitate their enhanced uptake into cells to overcome the drug resistance in MCF-7/ADR cells [168]. Similarly, Mao and co-workers found that PDPA-b-P(FPMA-co-OEGMA) nanomicelles conjugated with doxorubicin have either a negative or positive surface charge depending upon the tumor microenvironment and are able to efficiently deliver drugs to HeLa cells [169].

The tumor microenvironment is also responsible for creating the hypoxic conditions that lead to MDR, as oxygen-deprived cells grow slowly and are less susceptible to conventional chemotherapeutic drugs. Oxidative stress leads to changes in the cancer microenvironment. Increased oxidative stress promotes tumor development and associated drug resistance [170]. Targeting oxidative stress and the hypoxic microenvironment of tumors could also provide an opportunity to overcome MDR. Hypoxic conditions are associated with many cancers due to limited oxygen supply, which leads to overexpression of a transcription factor called HIF-1α. HIF-1α is the pivotal moderator of hypoxia-related responses that promote abnormal angiogenesis and MDR in several cancer subtypes [171]. The hypoxic conditions also critically influence the expression of ABC drug transporters [21, 165, 171]. Several studies have demonstrated that HIF-1α inhibition in cancer cells significantly sensitizes the cells to chemotherapeutic drugs and also contributes as an antagonist of p53-mediated cell death. Nanoformulations can easily target the HIF-1α factor to resume apoptotic signalling and contribute to the reversal of drug resistance. Tian et al*.* investigated the role of polymeric nanomaterials that mimic the cancer cell membrane and could be conjugated with haemoglobin and doxorubicin for reversal of drug resistance. The haemoglobin has an oxygen-carrying capacity that suppresses the expression of the HIF-1α factor, further downregulating the *MDR1* gene and enhancing cytotoxicity in MCF-7 and MCF-10A cell lines [172]. Yang et al*.* demonstrated the application of silver nitrate nanoparticles (AgNPs) to target angiogenesis by downregulating VEGF and GLUT1 gene expression and inhibiting HIF-1α signaling in MCF-7 cells [173]. Liu et al*.* evaluated the role of nanomicelles decorated with siRNA specific to silence the HIF-1α gene (siHIF) and doxorubicin in prostate cancer PC3 cells and in a xenograft mice model. Their findings indicated the inhibition of cell proliferation, disturbed angiogenesis and suppressed migration of cells in hypoxic conditions along with tumor growth inhibition in PC3 xenograft mice without elicitation of any immune reaction. siHIF-decorated nanomicelles downregulate *MDR1* gene expression and also sensitize the cells to doxorubicin under a hypoxic environment [174]. Lian et al*.* demonstrated the co-delivery of siHIF and cisplatin-conjugated chitosan-modified TPGS-b-(PCL-ran-PGA) nanoparticles in nasopharyngeal carcinoma for improved reversal of drug resistance in CNE-2 cells. The observations showed that silencing HIF-1α gene expression eventually inhibits P-gp expression, enhancing the sensitivity of cisplatin in multidrug-resistant cancer cells [175]. Song et al*.* observed that perfluorocarbon nanocarriers supply oxygen targeted to the tumor hypoxic microenvironment in tumor-bearing nude mice for lung re-oxygenation and to overcome drug resistance [176]. Alsaab et al*.* reported co-delivery of sorafenib and CA IX-C4.16 by TPGS nanoparticles in multidrug-resistant cancer cells to overcome hypoxia-mediated MDR. Sorafenib inhibited the p-AKT signaling pathway and upregulated the tumoricidal M1 macrophage by inducing caspase 3/7 apoptotic pathways in experimental human renal cell carcinoma A498/Evr resistant cells and RAW 264.7 macrophages [177].

### Nanomedical approaches to combat MDR mediated by dysfunctional cell cycle regulation

Cell cycle regulation is essential for proper cell division and growth; it is maintained and regulated by cyclins and cyclin-dependent kinases (CDKs). Some chemotherapeutic drugs specifically target different stages of the cell cycle to arrest the cell growth of rapidly dividing cancer cells. The overexpression of CDKs in cancer cells can also account for resistance to conventional chemotherapy [178]. A recent review published by Si et al*.* explains the crucial role of miRNA regulation in different types of cancer [179]. Polymeric nanosystem-mediated delivery of miRNA modulates CDK expression to overcome drug resistance. Co-delivery of miRNA with CDK inhibitors has a synergistic effect that enhances inhibition of tumor development and reversal of drug resistance. For example, Hallaj et al*.* showed the role of folic acid-conjugated chitosan nanoparticles for co-delivery of anti-CD73 siRNA and dinaciclib to manage tumor growth and to reverse drug resistance in murine breast cancer 4T1 cells, murine colon cancer CT26 cells and in xenograft mice [82]. Targeting the CDK4/6 cell cycle machinery using palbociclib and hydroxychloroquine-conjugated silica nanoparticles enhanced the biodistribution profile of chemotherapeutics and contributed to the reversal of MDR in pancreatic ductal adenocarcinoma in a xenograft mice model [180]. Deng et al*.* demonstrated the co-delivery of mir-34a and doxorubicin in hyaluronic acid-functionalized chitosan nanoparticles to target apoptotic signaling pathways. Their results demonstrated the enhanced delivery of a nanoformulation into tumor cells and inhibition of Bcl-2 expression and Notch-1 signaling pathways in human breast cancer MDA-MB-231 cells and in nude BALB/c mice [181]. Mittal et al*.* synthesized nanomicelles for co-delivery of gemcitabine and miRNA-205 in pancreatic cancer MIA PaCa-2^R^ and CAPAN-1^R^ cells and nude xenograft mice. Their results showed sustained drug release and miRNA-205-mediated suppression of tumor growth, activation of apoptosis-mediated signaling pathways and reversal of drug resistance [182]. Because nanoparticles are able to cross the blood–brain barrier, a siRNA-conjugated liposomal nanoformulation was used to overcome drug resistance in glioma CSCs. The glioblastoma cells typically have elevated levels of O^6^-methylguanine DNA methyltransferase (MGMT), a DNA repair protein that facilitates acquired drug resistance. In another study, Kato et al*.* synthesized a novel liposomal nanoformulation known as LipoTrust conjugated with siRNA that is able to silence the gene responsible for MGMT and enhance the sensitivity of glioma cells to treatment. Treatment with the nanoformulation led to a reduction in tumor volume and inhibition of the activity of the MGMT enzyme in a majority of the cells and in a xenograft mouse model [183].

### Nanomedical approaches to combat detoxification system-mediated MDR

Cytochrome P450 (CYP) superfamily enzymes oxidize fatty acids, steroids, and xenobiotics. They are involved in the clearance of various compounds from cells. In addition to carcinogenesis, CYP2 and CYP3 enzymes also contribute to MDR by activation or degradation of chemotherapeutic agents. There is a significant correlation between the upregulation of CYP enzymes and induction of the efflux transporters involved in the metabolism and detoxification of a wide spectrum of anticancer drugs, leading to MDR in cancer cells [184, 185]. For instance, the therapeutic effect of docetaxel was found to be constrained by CYP3A4/5 enzymes by oxidation to form pharmacologically inactive metabolites including t-butyl hydroxy docetaxel [186]. Other anticancer drugs such as paclitaxel, vincristine, teniposide, vinblastine, etc., are substrates of both CYP3A4 and P-gp [187–190]. Glutathione S-transferases (GSTs) also function along with efflux transporters where substrates or pharmacologically inactive metabolites conjugated with GSH tend to be effluxed by MRP transporters from the body [185]. A significant correlation has been established between CYP enzymes and drug efflux transporters. Hence, inhibition of CYP enzymes could also constitute an alternative therapy to overcome MDR [191]. Nanomaterials can actively modulate the regulation of CYP enzymes, serving as an anticancer therapy. Minko et al*.* demonstrated that doxorubicin conjugated with an HPMA copolymer has the potential to reverse drug resistance by inhibiting the drug detoxification system, inducing apoptosis by enhancing DNA damage and also suppressing UDP and glutathione expression [192]. Han and co-workers showed that inhibition of GST through ethacrynic acid-conjugated polymeric nanoparticles (MPEG-PLA-SS-ECA) could overcome the tumor cell detoxification system and associated drug resistance. Their findings demonstrate enhanced delivery of ethacrynic acid and inhibition of GST in cell lines. Two modifications were prepared for the purpose of disrupting the tumor detoxification system and overcoming drug resistance in oral squamous carcinoma SCC15/CBP and SCC15/PYM resistant cells, pingyangmycin (MPEG-PLA-SS-ECA/PYM) and carboplatin (MPEG-PLA-SS-ECA/CBP) [193]. Niu et al*.* developed organosilicate nanoparticles for co-delivery of ethacrynic acid (EA) and cisplatin to inhibit GST and intracellular GSH detoxification. The EA treatment induced inhibition of GST and enhanced intracellular uptake of cisplatin, synergistically preventing cellular detoxification in A375/DDP cells and suppressing tumor development in a nude xenograft murine model [194]. Wu et al*.* demonstrated the co-delivery of buthionine sulfoximine (a GSH inhibitor), celecoxib (a P-gp inhibitor) and doxorubicin in hybrid polymeric nanoparticles. They found that there was enhanced downregulation of GSH and P-gp expression and elevated intracellular doxorubicin retention in MCF-7/ADR cells. This nanomediated drug delivery platform exhibited the improved delivery of multiple target inhibitors and potent chemotherapeutic drugs to efficiently overcome MDR [78]. Zhu et al*.* developed cisplatin-conjugated 2-dimensional (2-D)-titanium carbide nanomaterials and evaluated their potency in non-small lung carcinoma A549/DDP-resistant cells and nude xenograft mice. Their results showed that the 2D nanomaterials interfered with total glutathione (GSH/GSSG) levels, expression of glutamylcysteine synthetase and glutathione peroxidase in both resistant cell lines and a murine model. The titanium carbide 2D nanomaterials also revealed excellent biocompatibility in a murine model, enhanced intracellular accumulation of cisplatin and suppression of tumor growth [195]. Wang et al*.* developed a glucosamine-grafted and doxorubicin-loaded hybrid nanosystem that interacted with GLUT1 receptors to enhance targeted receptor-mediated endocytosis in MCF-7 and MCF-7/ADR cells and tumor bearing nude xenograft mice. Due to elevated levels of GSH within cells, the pluronic L61 entity of the nanosystem induced intracellular ROS generation, release of cytochrome-c and also disruption of mitochondrial respiration. Intracellular doxorubicin accumulation was observed in cells that led to inhibition of cancer cell growth and tumor development, eventually facilitating the reversal of drug resistance [196]. Wang and co-workers demonstrated a long-term effect of copper nanoparticles (CuNPs) on CYP450 enzymes in rat brains. Their findings proved that a higher dose of CuNPs induces oxidative stress via hydroxyl radicals and malondialdehyde in the brain and a simultaneous decrease in the cellular intrinsic antioxidant enzyme system (total superoxide dismutase, glutathione). CuNPs also led to a reduction in the protein expression of CYP450 2C11/3A1 and eventually a reversal of the associated drug resistance in male rats [197].

### Nanotechnology-based approaches to combat MDR mediated by apoptotic pathways

Downregulation of apoptotic pathways is often observed in cancer cells. Activation of certain signal transduction pathways is known to result in decreased apoptotic cell death in cancer cells [7]. For example, upregulation of STAT family transcription factors plays a crucial role in cancer cell growth and metastasis, eventually leading to clinical MDR [198]. Also, a dysfunctional TP53 gene results in attenuated apoptosis in multidrug-resistant cancer cells [162, 199]. Engineered nanomaterial that targets multiple molecular pathways is an ideal therapeutic platform to eliminate MDR. Prabha et al*.* studied the efficacy of wild-type p53 DNA loaded into PLGA nanoparticles to treat breast cancer, and evaluated their antiproliferative activity. They found the stable and sustained transfer of the wild-type p53 gene into cells with antiproliferative activity to overcome drug resistance [200]. Choi et al*.* synthesized solid lipid nanoparticles for gene delivery to overcome drug resistance. Their results indicated efficient delivery of the p53 gene (plasmid DNA; pp53-EGFP) through nanoparticles in non-small cell lung carcinoma H1299 cells and in a xenograft murine model with improved biodistribution, inhibition of cell growth, suppression of tumor development and upregulated apoptotic pathways [201]. Wang et al*.* utilized the co-delivery of Bcl-2-specific siRNA and paclitaxel through a liposomal nanoformulation with the aim of silencing Bcl-2-mediated signaling pathways and suppression of tumor growth in human breast cancer MDA-MB-231 cells and in a 4T1 mouse model to facilitate the reversal of drug resistance [202]. Saad et al*.* also demonstrated the co-delivery of doxorubicin and siRNA specific to MRP1 and Bcl-2 mRNA via cationic liposomal nanoformulations to overcome drug resistance not related to drug efflux. Their results showed efficient drug accumulation, inhibition of efflux pumps via *MRP1* gene expression and induction of cell death mechanisms in human lung cancer H69AR cells, MCF-7/AD breast cancer cells, HCT15 colon cancer cells and A2780/AD ovarian cancer cells [203]. Chen et al*.* reported co-delivery of doxorubicin and Bcl-2 targeting siRNA via mesoporous silica nanoparticles resulting in enhanced cytotoxicity in human A2780/AD ovarian cancer cells. These nanoparticles bypassed efflux pumps and were internalized in perinuclear regions, thereby reversing drug resistance not dependent upon efflux pumps [204]. In another study, Fan and co-workers investigated folic acid-conjugated chitosan nanomicelles for the co-delivery of pyrrolidinedithiocarbamate (PDTC) and doxorubicin to reverse drug resistance in HepG2 liver cancer drug-resistant cells. PDTC is a potent NF-κB inhibitor. After the nanomicelles were internalized within resistant cells, NF-κB signaling was blocked and intracellular doxorubicin delivery and retention were enhanced, further overcoming drug resistance [205].

### Nanotechnology-based approaches to combat tumor cell heterogeneity and cancer stem cell-mediated MDR

Tumor heterogeneity is a distinct phenomenon that is a major impediment to the treatment of cancer. Clonal and subclonal mutations are mainly responsible for the heterogeneity of tumors. Heterogeneity also occurs due to the self-renewal and differentiation properties of tumors [206]. Genetic and environmental factors are the main causes of tumor heterogeneity and progression. It has been observed that subclonal mutations are enhanced in patients who receive chemotherapy mainly because the clonal population is eliminated by chemotherapeutic drugs during the initial treatment [207]. The emergence of resistant subclones that appear after the initial treatment instigates tumor expansion and eventually recurrence of tumors in the patients [208]. Tumor heterogeneity influences chemotherapeutic sensitivity and stimulates MDR mechanisms. Researchers are actively exploiting various nanosystems designed to circumvent MDR mediated by tumor heterogeneity. Ling et al*.* investigated pH-sensitive magnetic iron oxide nanoparticles (PMNs) for the treatment of resistant heterogeneous tumors in-vivo*.* The PMNs were used for diagnosis of early stage resistant heterogeneous tumors. They allowed dual-modal tumor diagnosis via MRI and fluorescence imaging of tumors with diameters up to 3 mm. PMNs engineered to respond to pH conditions within the tumor could be an efficient treatment strategy to overcome MDR [209]. Liu and co-workers applied the CRISPR/Cas9-based nanosystem nano-Cas9 ribonucleoprotein system (nanoRNP) to effectively combat tumor heterogeneity-mediated MDR. The nanoRNP conjugated with single guide RNAs (sgRNAs) specifically targeted and disrupted STAT3 and RUNX1 expression, thereby inhibiting the heterogenous tumor populations in glioblastoma U87MG cells and in a xenograft model [210].

Cancer stem cells (CSCs) are groups of cells (small subpopulations less than 1%) within a tumor that are characterized by stem-cell-like properties such as the ability to self-renew and to differentiate, leading to heterogeneity and acquired resistance to chemo- and/or radiotherapy [164]. Most chemotherapeutic drugs target rapidly dividing cells and therefore do not affect dormant CSCs. Active DNA repair signaling contributes to acquired MDR mechanisms in CSCs. The Notch pathway, Wnt/β signaling and elevated expression of ABC drug efflux transporters allow increased survival, stability and the slow proliferation rate of CSCs [29]. After initial chemotherapy, resistant CSCs repopulate the tumor by self-renewal and generate highly differentiated subpopulations. Cell surface markers such as CD133 and CD44 have exclusively been associated with the CSC phenotypic characteristics in different cancer types [211, 212]. Precise targeting of CSCs with drugs for their elimination is urgently needed to manage cancer relapse and recurrence. Elimination of CSCs via nanoformulations is one promising approach to overcoming MDR. Mamaeva et al*.* targeted the Notch signaling pathway, which is a potential regulator of CSCs and facilitates cancer progression. A nanomediated strategy to block the Notch pathway could work efficiently against CSCs. γ-secretase inhibitors (GSIs) conjugated with MSNs have demonstrated significant blocking of the Notch signaling pathway and reduction in tumor growth in in-vivo xenograft model after oral delivery of nanoparticles [28, 213].

Nanoconjugated gene silencing strategies have also been employed to target MDR-specific genes and inhibit CSC-mediated drug resistance. In one study, a lipid-based nanoformulation for co-delivery of paclitaxel and siRNA targeting CD133+ cells was evaluated to target the specific subsets of cells that are responsible for drug resistance and progression of colon cancer. The nanoformulation was evaluated in CHOK1 cells and gene silencing via siMDR1 was performed in CD133+ HT-29 colon cells which exhibited efficient *MDR1* gene knockdown and enhanced intracellular retention of paclitaxel and associated antitumor potency in colon cancer CSCs [214].

Tissue transglutaminase (TG-2) is a multifunctional enzyme and another key regulator that has a crucial role in CSC-mediated cancer progression and drug resistance. Verma et al*.* targeted the TG2 enzyme via co-delivery of gemcitabine and a siRNA-conjugated liposomal nanoformulation in pancreatic ductal adenocarcinoma (PDAC) nude mice. Their results showed efficient downregulation of endogenous TG2 by siRNA, inhibiting the growth of PDAC and further enhancing therapeutic antitumor activity [215]. Barth and co-workers synthesized indocyanine green (ICG)-conjugated calcium phosphosilicate nanoparticles (ICG-CPS NPs) for diagnostic imaging and drug delivery to CSC-mediated drug-resistant cancers. CD117 antigens are found abundantly on leukemia stem cells, so the ICG-CPS nanoparticles were decorated with anti-CD117 mAbs for direct targeting of NPs to CD117+ leukemia stem cells. The nanoformulation was found to mediate the elimination of specific leukemic cell populations responsible for drug resistance and disease progression in human samples as well as in a C3H/HeJ murine leukemia model [216].

## Conclusion and future prospective

Nanomaterials offer an extraordinary platform to overcome the limitations imposed by different mechanisms involved in the development of MDR. Combining conventional treatments with current nanotechnology advances might be a promising therapeutic approach to eliminate multidrug-resistant cancer. Nanomaterials are able to block P-gp and ABCG2 pumps and/or bypass the transporters to reverse drug-efflux-mediated MDR. Furthermore, nanomaterials functionalized with different targeting ligands allow therapeutic drugs to reach tumor sites directly via blood circulation. pH-sensitive nanosystems can exploit hypoxic tumor microenvironments, thereby reducing the expression of pro-angiogenic factors via downregulating the expression of HIF-1α. Stimuli-responsive nanosystems take advantage of unique cellular properties including pH variation, redox potential as well as enzymatic activation to overcome the MDR phenomena. Thermal, magnetic and light-based nanosystems have recently been identified for efficient reversal of drug resistance. Antibody-functionalized metal nanosystems can actively target and recognize multidrug-resistant tumor cells. Nanoparticles functionalized with siRNA are able to reprogram the gene expression pattern of resistant cells. Furthermore, nanomaterials improve the therapeutic specificity and enhance the biodistribution and pharmacokinetics of chemotherapeutic drugs. There are currently many nano-based formulations in clinical trials, and some are now used in the clinic. The merits of nanosystems need to be further explored to effectively combat drug-resistant cancer.

## Data Availability

Not applicable.

## Abbreviations

ADDC: Amphiphilic drug-drug conjugate
AgNPs: Silver nanoparticles
AOT: Aerosol OT (sodium bis(2-ethylhexyl)) sulfosuccinate
AuNPs: Gold nanoparticles
AuNRs: Gold nanorods
*β*-CD: Beta cyclodextrin
CdTe: Cadmium telluride
CuO: Copper oxide
DMA: N^6^-Carbobenzyloxy-l-lysine
3,6-dimethyl-1,4-dioxite-2,5-dione,DSPE-PEG: 1,2-Distearoyl-*sn*-glycero-3-phosphoethanolamine-poly(ethylene glycol)
DSPG: 1 2-Distearoyl-sn-glycero-3-phosphoglycerol
FA: Folic acid
HPMA: *N*-2(2-Hydroxypropyl) methacrylamide
Fe_3_O_4_: Iron oxide
mPEG: Poly(ethylene glycol) monomethyl ether
MPA: 3-Mercaptopropionic acid
MWCNTs: Multiwalled carbon nanotubes
NIPMAm: *N*-Isopropylacrylamide
NPs: Nanoparticles
OA: Oleic acid
P(ALA): Poly(aspartic-lipoic acid)
PBAE: Poly(beta-amino esters)
PBE: Poly butyl ether
PBCA: Poly(butyl cyanoacrylate)
PCL: Poly(epsilon-caprolactone)
PEO: Poly(ethylene oxide)
PEG: Polyethylene glycol
PEI: Polyethylenimine
P(OEGMA 300): Poly (oligo [ethylene glycol] methyl ether methacrylate
PGA: Poly(glycolic acid)
P(HEMA): Poly (2-hydroxyethyl methacrylate)
Poly-Jug-DA-b-PEG: Poly-juglanin-dithiodipropionic acid-b-poly ethylene glycol
PHB: Polyhydroxybutyrate
PLA: Poly lactic acid
PLL: Poly l-lysine
PLGA: Poly(lactic-co-glycolic) acid
QDs: Quantum dots
P-gp: P-glycoprotein
ROS: Reactive oxygen species
Pba: Phephobide a
SPION: Superparamagnetic iron oxide nanoparticles
TAT: Trans-Activator of Transcription
TPGS: Tocopheryl polyethylene glycol succinate
TCaNG: Tumor targeting Calcium ion nanogenerator
ZnO: Zinc oxide
